# Modeling the microbial pretreatment of camelina straw and switchgrass by *Trametes versicolor* and *Phanerochaete chrysosporium via* solid-state fermentation process: A growth kinetic sub-model in the context of biomass-based biorefineries

**DOI:** 10.3389/fmicb.2023.1130196

**Published:** 2023-04-06

**Authors:** Cuong Ngoc Dao, Lope G. Tabil, Edmund Mupondwa, Tim Dumonceaux

**Affiliations:** ^1^Department of Chemical and Biological Engineering, University of Saskatchewan, Saskatoon, SK, Canada; ^2^Agriculture and Agri-Food Canada, Saskatoon Research Centre, Saskatoon, SK, Canada

**Keywords:** biofuel, fungal pretreatment, solid-state fermentation, mathematical modeling, camelina, switchgrass

## Abstract

Advancing microbial pretreatment of lignocellulose has the potential not only to reduce the carbon footprint and environmental impacts of the pretreatment processes from cradle-to-grave, but also increase biomass valorization, support agricultural growers, and boost the bioeconomy. Mathematical modeling of microbial pretreatment of lignocellulose provides insights into the metabolic activities of the microorganisms as responses to substrate and environment and provides baseline targets for the design, development, and optimization of solid-state-fermentation (SSF) bioreactors, including substrate concentrations, heat and mass transfer. In this study, the growth of *Trametes versicolor* 52J (TV52J), *Trametes versicolor* m4D (TVm4D), and *Phanerochaete chrysosporium* (PC) on camelina straw (CS) and switchgrass (SG) during an SSF process was examined. While TV52J illustrated the highest specific growth rate and maximum cell concentration, a mutant strain deficient in cellulose catabolism, TVm4D, performed best in terms of holocellulose preservation and delignification. The hybrid logistic-Monod equation along with holocellulose consumption and delignification models described well the growth kinetics. The oxygen uptake rate and carbon dioxide production rate were directly correlated to the fungal biomass concentration; however, a more sophisticated non-linear relationship might explain those correlations better than a linear model. This study provides an informative baseline for developing SSF systems to integrate fungal pretreatment into a large-scale, on-farm, wet-storage process for the utilization of agricultural residues as feedstocks for biofuel production.

## 1. Introduction

Bioenergy demonstrates the best life-cycle performance in terms of greenhouse gas emissions mitigation when wastes and residues are exploited, which avoids the impacts of dedicated crop production and waste management. Therefore, bioenergy is considered in all of the climate change mitigation scenarios to decarbonize the energy system (Edenhofer, 2015; Daioglou et al., 2020). Pretreatment is a crucial step in both biofuel pellets and bioethanol production to enhance the binding mechanisms or enzyme substrate accessibility (Bhatia et al., 2020; Robak and Balcerek, 2020). Pretreatment employing white-rot fungi, which produce the high-redox-potential ligninolytic peroxidases (lignin peroxidase–LiP, manganese peroxidase–MnP, versatile peroxidases–VP) and low-redox-potential laccase was observed to improve the physical-mechanical properties of the pelletized materials as well as enhance their enzymatic digestibility for bioethanol production (Gao et al., 2017; Kalinoski et al., 2017; Onu Olughu et al., 2021). Compared to other conventional pretreatment methods, biological pretreatment possesses several advantages including 1) an environmentally-friendly process, 2) moderate operating conditions, 3) higher productivity, 4) potential for value-added by-products, 5) fewer fermentation-inhibiting compounds, 6) few side reactions, 7) lower energy consumption, 8) lower requirements for reactor resistance to pressure and corrosion (Chen et al., 2010; Wan and Li, 2012; Agrawal et al., 2015; Su et al., 2018; Sharma et al., 2019) (Supplementary Table 1). Combining microbial pretreatment with conventional processes can positively impact the required severity of the pretreatment (Shirkavand et al., 2016). Advancing fungal/microbial pretreatment techniques will not only reduce the carbon footprint and environmental impacts of the pretreatment process from cradle-to-grave, but will also increase the valorization of lignocellulosic biomass, support agricultural growers, and develop the bioeconomy.

*Trametes versicolor* (TV52J) and *Phanerochaete chrysosporium* (PC) are white-rot fungi that have been thoroughly examined in several applications including bio-degradation (Machado et al., 2020; Tišma et al., 2021). They were observed to adequately break down and partially degrade lignin to facilitate the consequent enzymatic hydrolysis and fermentation (Dashtban et al., 2010; Sánchez and Montoya, 2013; Bajpai, 2016). *T. versicolor* degrades lignocellulose partly by oxidizing the lignin using several secreted oxidative and peroxidative enzymes (De Jong and Field, 1997) and its delignification is faster than that of other organisms due to the activities of lignin peroxidase (LiP), manganese peroxidase (MnP), and laccase (Uzun et al., 2021). An engineered mutant strain of *T. versicolor*, TVm4D, which lacks cellobiose dehydrogenase (CDH), is unable to access and utilize crystalline cellulose as a carbon source, yet preserves the lignin-degrading capabilities of the wild-type parent strain (Canam et al., 2011; Ramirez-Bribiesca et al., 2011). CDH, an extracellular oxidoreductase, oxidizes cellobiose and produces free radicals that can attack and degrade cellulose (Phillips et al., 2011). Therefore, TVm4D may have the capacity to increase carbohydrate availability through the degradation of lignin while restricting its utilization of cellulose. *P. chrysosporium* is a common white-rot fungus that utilizes plant cell wall carbohydrates as a carbon and energy source while secreting ligninolytic enzymes for delignification (Sun et al., 2022).

The two feedstocks considered in this study were camelina straw and switchgrass. Camelina [*Camelina sativa* (L.)] is a potential niche-filling crop that can be used in biofuel production (Krohn and Fripp, 2012). It is a spring annual oilseed plant that grows well under low temperatures and drought conditions, is relatively resistant to plant pathogens and insect pests, matures faster than other oilseed crops (85–100 d), requires fewer agro-inputs, and is compatible with present farm equipment (Krohn and Fripp, 2012; Ibrahim and El Habbasha, 2015). The production of Camelina-derived biodiesel yields a net energy ratio of 1.47 and its utilization releases fewer emissions than diesel fuel in different scenarios (Patil and Deng, 2009; Bacenetti et al., 2017). The leftover residue from camelina harvesting (camelina straw, **CS**) can be considered as a feedstock for the production of biofuel pellets and/or bio-ethanol. Switchgrass (*Panicum virgatum*, **SG**) is a high-yielding perennial grass species that is native to North America and is assigned as an energy crop by the U.S. Department of Energy due to its high biomass yield, compatibility with conventional farming practices, cold/drought tolerance (upland ecotypes), minimal fertilizer input requirement, and high hemicellulose content (Adler et al., 2006; Colley et al., 2006). Switchgrass is favorable for pellet production thanks to its higher throughput rate and lower drying energy as compared to woody biomass (Samson et al., 2000). It is also considered a promising energy crop to produce biofuels such as ethanol (Kaliyan and Morey, 2009).

Despite the great potential for using solid-state fermentation (SSF)-based biological pretreatment for biofuel production, there are many obstacles to overcome to provide a feasible process on an industrial scale, including the long pretreatment time (Sathendra et al., 2022). In the attempt to scale up the integration of microbial pretreatment into the fuel production route, large-scale on-farm-indoor-wet-storage fungal pretreatment, combined with on-farm densification, is believed to lessen the drawback of long pretreatment time, improve the fuel's density, and therefore reduce the associated cost and carbon footprint of transporting the fuel to its destination. However, the scarcity of realistic and scalable SSF bioreactor designs supported by accurate and reliable mathematical models and fully automatic control systems that could lessen the impact of the heterogeneity of heat and mass transfers discourages the application of that concept. Modeling microbial growth during the solid-state fermentation (SSF) process is a powerful tool to design and develop SSF bioreactors and forecast and optimize their operating performance at the production scale (Mitchell et al., 2006b). A successful model representing the biological pretreatment system will provide quick monitoring or prediction of holocellulose and lignin content, which are important indices of biofuel production, or oxygen and moisture requirement, which are essential for maintaining or stopping the microorganisms, or carbon dioxide production, a greenhouse gas, which is important for the life-cycle assessment of the process. There have been a few attempts to model the interactions of white-rot fungi with lignocellulosic substrates in which logistic and Monod models were heavily used (Kazartsev and Soloviev, 2012; Shi et al., 2012, 2014; Montoya et al., 2014, 2015; Olorunnisola et al., 2018; Sánchez and Montoya, 2020).

The purpose of this study was to investigate the growth of *T. versicolor* m4D (TVm4D), *T. versicolor* 52J (TV52J), and *P. chrysosporium* (PC) on camelina straw (CS) and switchgrass (SG). The study focused on assessing fungal biomass accumulation, substrate consumption, O_2_ uptake, CO_2_ generation, and enzyme production. The study developed mathematical models to gain insights into the growth kinetics of the fungi. Growth-associated parameters were evaluated to monitor holocellulose and lignin content, cumulative oxygen consumption, carbon dioxide production, and enzyme concentration as functions of pretreatment time. Environmental factors such as temperature, humidity, and pH were not investigated in this study. Furthermore, the developed model may not be applicable to other fungal species or substrates, as each system has unique characteristics that may require a different approach. Mass and energy balances and transport circumstance sub-model were beyond the scope of this study. The results of this study can be useful for the life-cycle assessment of biofuel-tailored microbial pretreatment.

## 2. Materials and methods

### 2.1. Feedstock preparation

Camelina straw (CS) was supplied by the AAFC Research Farm (Saskatoon Research and Development Centre, SK, Canada). Camelina was a replicated trial that mixed 17 different breeding lines plus the cultivars Midas, Cypress, Sonny, Dolly, Calena, and AAC 10CS0046. The camelina was seeded in May and harvested in September 2021. The straw was left to dry on the field and collected manually into the cloth bags. Switchgrass (SG) used in this study was the “Cave-in-rock” variety. SG was harvested in October 2021 in Nappan, Nova Scotia using a disk mower. It remained in a swath in the field for at least a week. The feedstock was randomly gathered from the swath and spread in a storage building for 2 months to dry. The initial moisture content of CS and SG were examined by following ASABE (2016). The feedstocks were dried at 40°C for 72 h and then ground to pass through 3-mm and 1-mm sieve using a laboratory knife mill (SM1, Retsch Technology GmbH, Haan, Germany) for microbial pretreatment and compositional analysis, respectively. The ground feedstock (about 5% moisture content) was stored in zip lock bags at −20°C before use.

### 2.2. Fungal strain and liquid inoculum preparation

The white-rot fungi used in this study were 1) *T. versicolor* m4D, a cellobiose dehydrogenase (CDH)-deficient strain (mutant) of *T. versicolor* (Canam et al., 2011); 2) *T. versicolor* 52J (ATCC 96186); and 3) *P. chrysosporium*. These three strains were kept as glycerol stocks frozen at −80°C and were cultivated on Difco malt extract agar (MEA) (Benton Dickenson, Sparks, MD). Ten agar plugs (5 mm in diameter) were removed from the edge of the colony grown on the malt extract agar plate and put into 250 ml of a 2% malt extract broth (MEB) by using the wide end of a sterilized Pasteur pipette. This mixture was blended in a sterilized Eberbach blender cup (Eberbach, Charter Township, MI, USA) at full speed on a Waring blender base (Waring Lab, USA) (3 pulses of 10 s), and then the blended mixture was poured into a 500 ml cotton-plugged Erlenmeyer flask and incubated at 28°C with 150 rpm of rotating shaking for 7 d. Before being used for inoculum, the liquid culture was again homogenized using a sterilized Eberbach blender cup on a Waring blender base for three consecutive 10-s cycles.

### 2.3. Solid-state fermentation of lignocellulosic substrates

**Figure 1:**
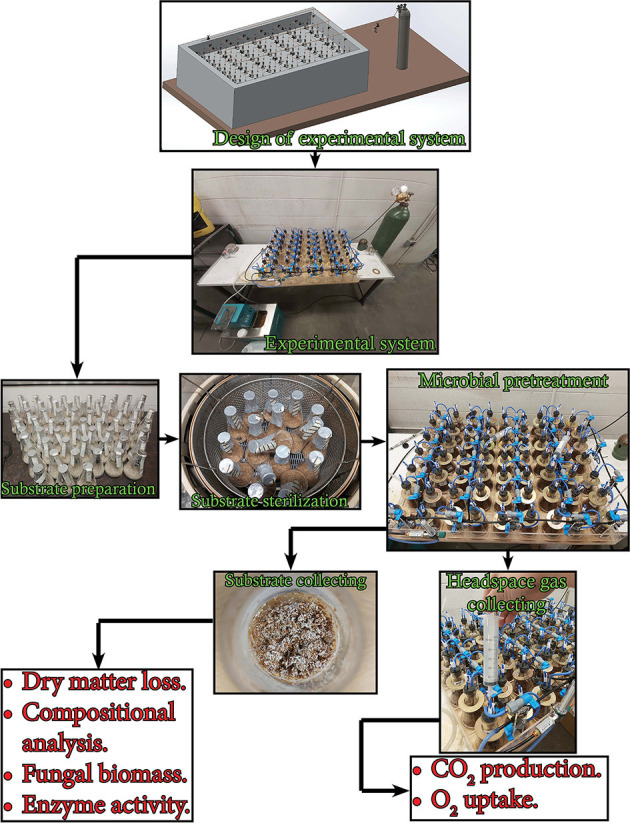
Flow diagram of the experiment in this study indicating the measurements undertaken.

The experiment used in this study is illustrated in Figure 1. The experimental setup consisted of 21 units. Each unit contained three 250-ml Erlenmeyer flasks sealed with customized rubber stoppers supporting gas inlet and outlet ports with clips and inline 0.33 μm sterile gas filters. The gas inlet port was connected to the main distribution tube *via* a stop valve. The main distribution tube was connected to an oxygen tank *via* a 0.2 μm sterile filter, a control valve, and a gas flowmeter. All flask units were submerged in a water bath using copper flanges. The water temperature was maintained by a refrigerated/heating bath circulator (RW-0525G model, Jeiotech Lab Companion, Yuseong-gu, Daejeon, Korea). Three grams of the substrate (dry basis) was moisturized by a predetermined amount of distilled water in a 250-ml Erlenmeyer flask to achieve a final moisture content of 75% (wet basis) and then the flask was autoclaved at 121°C for 30 min. The cool moist substrate in the flask was inoculated with 2 ml of blended fungal liquid pre-culture (prepared as in Section 2.2) in a biological safety cabinet. Controls (no fungal inoculum) were prepared by adding 2 mL of sterile distilled water instead of the blended fungal liquid culture. The pretreatment was carried out in the water bath at 28°C for 30 d. Every 24 h, the headspace gas of the flask was collected and all the flasks were flushed with pure oxygen with a flow rate of 125 ml min^−1^ for 5 min. The flasks were also sampled at days 0, 2, 4, 6, 8, 10, 12, 14, 18, 22, 26, and 30 to collect the substances for the determination of fungal biomass, holocellulose, and lignin content.

### 2.4. Analytical methods

#### 2.4.1. Headspace gases analysis

The headspace gas was collected by a 20 ml syringe equipped with a clip and a stop valve. Then it was injected into a 12-mL Exetainer vial *via* a needle. Gas analyzes were conducted following the protocol provided by Agilent (2012). The exetainer vials were connected to an autosampler (CombiPAL, Agilent Technologies, CA, USA) integrated with a gas chromatography analyzer (490 Micro GC System, Agilent Technologies, Santa Clara, CA, USA) equipped with 3 channels including channel 1: Agilent J&W CP-Molsieve 5 molecular-sieve-coated PLOT capillary column; channel 2: Agilent J&W PoraPLOT U Column; and channel 3: CP-Sil 5 CB Column, with the corresponding column temperature/pressure of (80°C, 200 kPa), (80°C, 150 kPa), and (60°C, 150 kPa) with ultra-high purity 5.0 argon and helium as carrier gases (35 ml min^−1^ of flow rate), respectively. All channels were connected to a thermal conductivity detector (TCD) (working temperature 90°C) and the run time was 1 min for O_2_, CO_2_, and N_2_. The rate of O_2_ consumption and CO_2_ production were determined by Equation (1) (Smits et al., 1996):


(1) $$\begin{array}{l} r_{\text{i}}=\frac{\Delta c_{\text{i}}}{\Delta t}\frac{VP}{RT}{C_{\text{i}}}^{\text{air}}\frac{1}{m_{\text{S}}} \end{array}$$

where:

*r*_i_ = the rate of O_2_ consumption or CO_2_ production, [mmol (g d.b. substrate)^−1^ d^−1^];

Δ*c*_i_ = the decrease of O_2_ concentration or increase of CO_2_ concentration in term of volume percentages over the specific period Δ*t*, [% v/v];

Δ*t* = the time gap between two measurements, [d];

*V* = the gas volume in the system after being corrected for the volume captured by the substrate, [L];

*P* = the atmospheric pressure, *P* = 1 [atm];

*R* = the ideal gas constant, *R* = 0.082057 [L atm (mol)^−1^ K^−1^];

*T* = the incubation temperature, *T* = 301 [K];

$C_{\text{i}}^{\text{air}}=$ the specific gas concentration of flushing gas, since it was pure oxygen therefore $C_{\text{i}}^{{\text{O}}_{2}}=100\%=1$;

*m*_S_ = mass of solid substrate in dry basis, [g].

#### 2.4.2. Holocellulose and lignin content

Cellulose, hemicellulose, and lignin content were determined based on the two-step acid hydrolysis based on the NREL Laboratory Analytical Procedure (LAP) (Sluiter et al., 2005, 2010). An amount of 300 ± 10 mg of extractive-free, 2-mm-knife mill-ground, No. 80 (180 μm)-sieve-retained substrate was hydrolyzed by 72% (w/w) H_2_SO_4_ for 1 h at 30°C and then by 4% (w/w) H_2_SO_4_ at 121°C for 1 h. Acid-insoluble lignin (AIL) was determined by gravimetric analysis while the acid-soluble lignin was monitored by a UV-Visible spectrophotometer (UV mini-1240, Shimadzu Corp., Kyoto, Japan) at 240 nm of absorbance wavelength. Cellulose and hemicellulose, which were acid-hydrolyzed into monomeric sugars, were determined by HPLC using a Biorad Aminex HPX-87P column and a refractive index detector (RID). The HPLC conditions were 1) 10–50 μL of injection volume, 2) 0.2 μm filtered and degassed HPLC grade water as the mobile phase, 3) 0.6 ml min^−1^ of flow rate, 4) 80–85°C of column and detector temperature, and 5) 35 min of run time. Cellulose was determined by glucose while hemicellulose was estimated from xylose, arabinose, galactose, and mannose, with 0.90 and 0.88 of anhydro correction for C-6 and C-5 sugars, respectively.

#### 2.4.3. Fungal biomass content

Ergosterol content has been widely used to determine the fungal biomass concentration on lignocellulosic substrates (Hobbie et al., 2009; Canam et al., 2011). The microbially pretreated sample (200–400 mg, d.b.) was heated in 5 ml of methanol containing 5% KOH at 80°C for 1 h. The medium was then liquid-liquid extracted with hexane three times, and then the Hexane aliquots were combined, evaporated to dryness, and then re-dissolved in methanol. The solution was then filtered through a 0.22 μm nylon filter into a 2.0 ml HPLC vial. The sample was analyzed by an HPLC system comprising a multi-solvent delivery system (600E, Waters Corporation, Milford, MA, USA) integrated with an in-line degasser, a 25 μL loop equipped-Rheodyne injection valve (Interchim, Montluçon, France), a UV detector (SPD-6AV, Shimadzu Corporation, Kyoto, Japan), and a Chromatopac integrator (CR-3A, Shimadzu Corporation, Kyoto, Japan). The column was a LiChrospher^®^ RP-18 HPLC Column (5 μm particle size, L × I.D. = 25 cm × 4.6 mm, end-capped, Merck Group, Darmstadt, Germany) protected by a Spherisorb C18 guard column (Waters Corporation, Milford, MA, USA). Methanol (98% methanol/2% water) eluant as a mobile phase had a flow rate of 1.5 ml min^−1^ with the column temperature upheld at 28°C. The wavelength of UV detection was 282 nm (Ravelet et al., 2001). Pure fungal mycelium was prepared by cultivating a 0.5-cm-diameter agar plug of a fully colonized PDA plate on another PDA plate covered by a 0.1 μm pore size polycarbonate membrane filter (Sterlitech) for 7 d at 28°C. The pure fungal layer was then removed from the membrane and mixed with the lignocellulosic substrate with different fungal biomass/substrate biomass ratios to determine the correlation between the ergosterol content and the fungal biomass/substrate biomass ratio.

#### 2.4.4. Enzyme production

All secreted fungal enzymes extracted from the solid culture, including lignin peroxidase, manganese peroxidase, versatile peroxidase, and laccase, as well as cellulases and hemicellulases, were counted in the enzyme production model. The enzyme concentration (*P*) was determined based on the total Kjeldahl nitrogen (TKN) measured in the filtered enzyme extracts. The cultivated solid substrate (dry basis) was mixed with sodium acetate buffer (30 ml, pH 4.5, 0.05 M) and incubated for 30 min at 39°C. The suspension was then filtered through a crucible (30 ml–30F Kimax, Thermo Fisher Scientific, Waltham, MA, USA). The filtered solution was filtered a second time *via* a 0.7 μm glass microfiber filter (Type GF/F, Whatman Inc., Florham Park, NJ) and frozen at −80°C before TKN analysis. The total protein was the product of protein concentration and extracted enzyme total volume (Wan, 2011).

### 2.5. Data analysis and parameter estimation

#### 2.5.1. Growth of fungal biomass

Linear, exponential, logistic, deceleration, Tessier, and Monod equations have been employed for modeling the fungal cell biomass growth on SSF (Sangsurasak et al., 1996; Mitchell and Krieger, 2006a; Wan, 2011; Kazartsev and Soloviev, 2012; Fathima et al., 2013; Shi et al., 2014; Sánchez and Montoya, 2020). However, the logistic model (Equation 2) and Monod model (Equation 3) have been employed widely to examine the fermentation process, investigate microbial consortia interactions, and explore unknown biological phenomena (Fuad, 2020; Xu, 2020). While the logistic model is convenient to quickly examine the growth (Viccini et al., 2001; Lenz et al., 2004; Mitchell and Krieger, 2006a), it only considers the biomass concentration and ignores the substrate utilization (Kargi, 2009). Unlike the logistic model which is purely based on some mechanistic fundamentals of biological population growth, the Monod model is rigidly empirical and intended to fit experimental data of cell growth rate vs. growth-limiting substrate concentration (Liu, 2007; Fuad, 2020). In this study, a hybrid logistic-Monod model (Equation 4) was employed to consider both substrate-limiting and self-inhibiting factors (Xu, 2020; Li et al., 2022; Tsafrakidou et al., 2022). In this hybrid model, fungal biomass accumulation and substrate degradation initially proceeded through the substrate-limiting stage indicated by the Monod equation and progressively shifted toward the self-inhibiting stage characterized by the logistic equation.


(2) $$\begin{array}{l} \frac{\text{d}X}{\text{d}t}=\mu_{\text{m}}X\left(1-\frac{X}{X_{\text{m}}}\right) \end{array}$$

(3) $$\begin{array}{l} \frac{\text{d}X}{\text{d}t}=\mu X=\frac{\mu_{\text{m}}SX}{K_{\text{S}}+S} \end{array}$$

(4) $$\begin{array}{l} \frac{\text{d}X}{\text{d}t}=\mu_{\text{m}}\left(\frac{S}{K_{\text{S}}+S}\right)\left(1-\frac{X}{X_{\text{m}}}\right)X \end{array}$$

where:

*X* = fungal biomass concentration, [g X (g d.b. substrate)^−1^];

μ = specific growth rate, [d^-1^];

μ_m_ = maximum specific growth rate, [d^-1^];

*t* = incubation time, [d];

*S* = substrate concentration, [g holocellulose (g d.b. substrate)^−1^];

*K*_s_ = half saturation coefficient, the value of *S* when μ = 0.5μ_m_.

*X*_m_ = maximum fungal biomass concentration, also known as a self-inhibiting factor, [g X (g d.b. substrate)^−1^].

#### 2.5.2. Oxygen consumption rate

The expression for O_2_ consumption within an O_2_ balancing environment is given by Equation (5) (Mitchell and Krieger, 2006b; Shi et al., 2012, 2014):


(5) $$\begin{array}{l} \text{OUR}=\frac{\text{d}{\text{O}}_{2}}{\text{d}t}=\frac{1}{Y_{\text{X}/\text{O}}}\frac{\text{d}X}{\text{d}t}+m_{\text{O}}X \end{array}$$

where:

OUR = oxygen uptake rate, [mmol d^−1^];

*Y*_X/O_ = yield coefficient from oxygen to fungal biomass, [g X (mmol O_2_)^−1^];

*m*_O_ = maintenance coefficient, [mmol O_2_ (g X)^−1^ d^−1^].

#### 2.5.3. Carbon dioxide production rate

The indication of CO_2_ evolution inside a CO_2_ balancing environment is given by Equation (6) (Mitchell and Krieger, 2006b):


(6) $$\begin{array}{l} \text{CPR}=\frac{\text{d}{\text{CO}}_{2}}{\text{d}t}=Y_{\text{C}/\text{X}}\frac{\text{d}X}{\text{d}t}+m_{\text{C}}X \end{array}$$

where:

CPR= carbon production rate, [mmol d^−1^];

*Y*_C/X_ = yield of CO_2_ from fungal biomass, [mmol CO_2_ (g X)^−1^];

*m*_C_ = maintenance coefficient, [mmol CO_2_ (g X)^−1^ d^−1^].

#### 2.5.4. Carbohydrate consumption and delignification

In this study, it was assumed that energy from the delignification was too small to be accounted for part of the energy utilization for microbial growth and holocellulose was the sole carbon and energy source to support the metabolism for biomass growth and provided the energy for delignification (Wan, 2011; Shi et al., 2012). In Equation (7), holocellulose was spent for fungal growth (the first component in the equation), maintenance of microbial cells (the second component), and providing energy for the delignification (the third component).


(7) $$\begin{array}{l} \frac{\text{d}S}{\text{d}t}=-\frac{1}{Y_{\text{X}/\text{S}}}\frac{\text{d}X}{\text{d}t}-m_{\text{C}}X-k_{\text{LD}}\frac{\text{d}L}{\text{d}t} \end{array}$$

where:

*S* = holocellulosic substrate concentration, [g holocellulose (g d.b. substrate)^−1^];

*Y*_X/S_ = yield coefficient from substrate to fungal biomass, [g X (mmol O_2_)^−1^];

*m*_C_ = maintenance coefficient, [g S (g X)^−1^];

*k*_LD_ = lignin degradation coefficient;

*L* = lignin concentration, [g lignin (g d.b. substrate)^−1^].

Lignin degradation was assumed to be a first-order, non-growth-associated reaction (Equation 8). The degradation coefficient, *k*_l_, was proposed to be an exponential function of decay time (Equation 9). Therefore, the decomposed model (Equation 10) was expected to accurately predict the experimental data of lignin degradation. The derivative form of Equation (10) was expressed in Equation (11):


(8) $$\begin{array}{l} -r_{\text{L}}=-\frac{\text{d}L}{\text{d}t}=k_{\text{l}}L \end{array}$$

(9) $$\begin{array}{l} k_{\text{l}}=k_{\text{l}}\left(t\right)=k_{1}e^{-k_{2}t} \end{array}$$

(10) $$\begin{array}{l} L=L_{0}e^{\frac{k_{1}}{k_{2}}\left(e^{-k_{2}t}-1\right)} \end{array}$$

(11) $$\begin{array}{l} \frac{\text{d}L}{\text{d}t}=-e^{\frac{-1+e^{-k_{2}t}}{k_{2}}-k_{2}t}k_{1}L_{0} \end{array}$$

where:

*k*_1_ = the first lignin degradation constant;

*k*_2_ = the second lignin degradation constant;

*L*_0_ = the initial lignin concentration of the substrate, [g lignin (g d.b. substrate)^−1^].

#### 2.5.5. Enzyme production

Enzyme formation was modeled by Luedeking and Piret equation (Equation 12) (Luedeking and Piret, 1959; Garnier and Gaillet, 2015).


(12) $$\begin{array}{l} \frac{\text{d}P}{\text{d}t}=\alpha \frac{\text{d}X}{\text{d}t}+\beta X \end{array}$$

where:

*P* = enzyme concentration, [g proteins (g d.b. substrate)^−1^];

α = growth-associated constant for enzyme production, [g proteins (g X)^−1^];

β = non-growth-associated constant for enzyme production, [g proteins (g X)^−1^ d^−1^];

#### 2.5.6. Statistical analysis and parameter estimation

All combination treatments were carried out in triplicate. Each headspace gas sampling was conducted randomly twice. The models were built in derivative forms and numerically solved using R version 4.2.2 (2022-10-31) (R Core Team, 2022). The model parameters were estimated by nonlinear regression from the experimental data by using the ode and nls.lm functions. The function ode, a general solver for ordinary differential equations, was acquired from the package deSolve (Soetaert et al., 2010). The function nls.lm, provided by minpack.lm package, addressed nonlinear least-squares problems with the Levenberg-Marquardt algorithm (Elzhov et al., 2016). The Levenberg-Marquardt algorithm integrates two numerical minimization algorithms: 1) the Gauss-Newton method and 2) the gradient descent method. In the Gauss-Newton method, the sum of squared errors is decreased by presuming the least squares function is regionally quadratic in the parameters and searching for the least value of this quadratic. In the gradient descent method, the sum of square errors is declined by continuously revising the parameters in the steepest-descent direction (Gavin, 2019). First, a function fn that generates a vector of residuals whose sum of squares is to be minimized, is defined. Then the nls.lm was used to minimize the sum square of the vector returned by the function fn by the Levenberg-Marquardt algorithm (Elzhov et al., 2016).

## 3. Results and discussions

### 3.1. Fungal biomass growth

**Figure 2:**
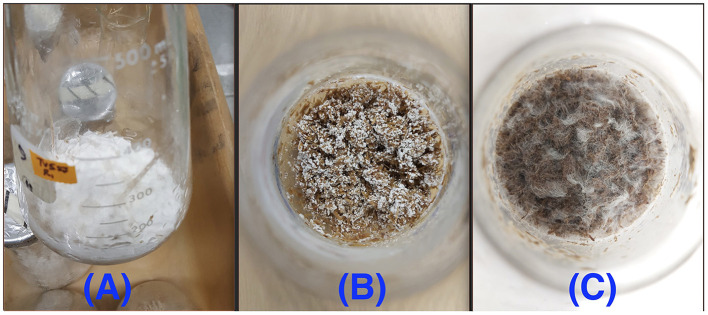
Fungal growth on the lignocellulosic substrates after 14 d: **(A)**: CS-TV52J, **(B)**: SG-TVm4D, **(C)**: CS-PC. PC, *P. chrysosporium*; TV52J, *T. versicolor* 52J; TVm4D, *T. versicolor* m4D; CS, camelina straw; SG, switchgrass.

**Figure 3:**
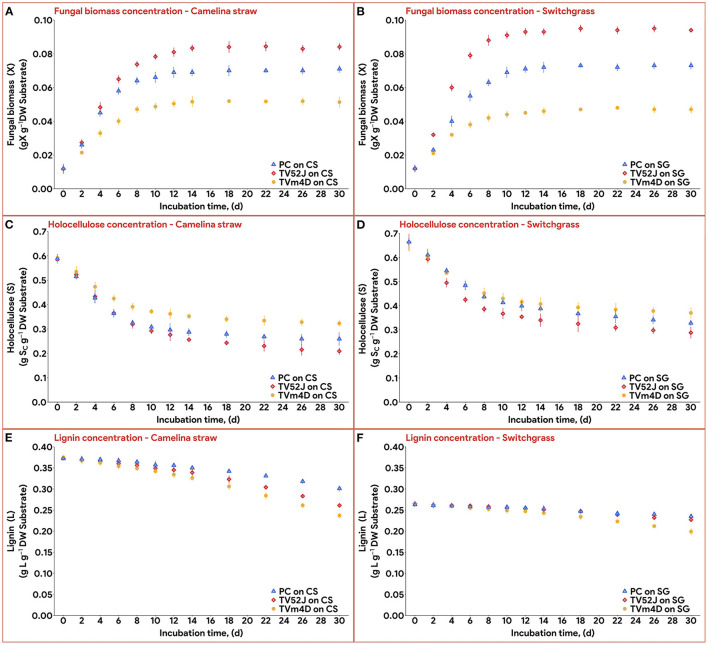
Concentration of fungal biomass (X), holocellulose substrate (S), and lignin content (L) during 30 d of microbial pretreatment: **(A)**: X profiles of CS, **(B)**: X profiles of SG, **(C)**: S profiles of CS, **(D)**: S profiles of SG, **(E)**: L profiles of CS, **(F)**: L profiles of SG. PC, *P. chrysosporium*; TV52J, *T. versicolor* 52J; TVm4D, *T. versicolor* m4D; CS, camelina straw; SG, switchgrass.

**Figure 4:**
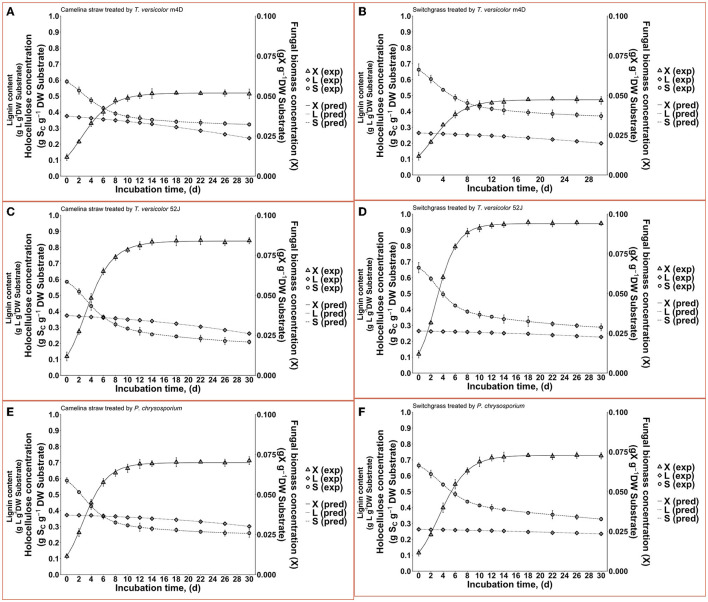
Time profiles of fungal biomass, holocellulose, and lignin concentration during 30 d of microbial pretreatment: **(A)**: profiles of TVm4D on CS, **(B)**: profiles of TVm4D on SG, **(C)**: profiles of TV52J on CS, **(D)**: profiles of TV52J on SG, **(E)**: profiles of PC on CS, **(F)**: profiles of PC on SG. X, fungal biomass; L, lignin; S, holocellulose; exp, observed data from the experiment; pred, predicted values calculated by the proposed models.

All three fungi grew well on both CS and SG. Cell concentrations of those fungi reached their limits after 14–22 d. The fungal mycelia occupied the whole surface area of the substrate inside the flask after 2 weeks (Figure 2). The time courses of fungal biomass generation, holocellulose consumption and lignin degradation during 30 d of microbial pretreatment are illustrated in Figure 3. TV52J showed the highest growth capacity, which was indicated by the greatest maximum fungal biomass concentration (Figures 3A, B) and holocellulose consumption (Figures 3C, D). As expected based on its impaired ability to metabolize cellulose, the mutant strain, TVm4D, performed the best in terms of holocellulose preservation (Figures 3C, D) and lignin degradation (Figures 3E, F). Maximum fungal biomass concentration was 0.084, 0.071, and 0.053 g X (g d.b. substrate)^−1^ for CS-TV52J, CS-PC, and CS-TVm4D, respectively, while those of SG-TV52J, SG-PC, and SG-TVm4D were 0.095, 0.074, and 0.048 g X (g d.b. substrate)^−1^, respectively (Figures 3A, B). The estimated maximum specific growth rate (μ_max_), half-saturated coefficient (*K*_s_), and self-inhibiting factor (*X*_m_) of each substrate-fungus combination are indicated in Table 1 and the plots of predicted values against the experimental data of the growths are shown in Figure 4.

**Table 1:** Estimated parameters of fungal growth, substrate consumption, oxygen uptake, carbon dioxide release, and enzyme production models.

**Growth kinetics**	**Parameter**	**Symbol**	**Unit**	**Treatment**					
				**CS-TVm4D**	**CS-TV52J**	**CS-PC**	**SG-TVm4D**	**SG-TV52J**	**SG-PC**
Fungal growth	Maximum specific growth rate	μ_max_	[d^−1^]	1.005	1.210	1.005	0.911	1.216	0.788
	Half saturation coefficient	K_s_	[d^−1^]	0.682	0.664	0.415	0.690	0.539	0.418
	Self inhibiting factor	X_m_	[g X (g d.b. substrate)^−1^ ]	0.052	0.084	0.070	0.047	0.094	0.073
Holocellulose consumption	Yield coefficient from substrate to biomass	*Y*_x/s_	[g X (g d.b. substrate)^−1^ ]	0.180	0.249	0.213	0.152	0.296	0.247
	Cell maintenance coefficient	m_s_	[g S (g X)^−1^]	0.086	0.081	0.059	0.100	0.085	0.046
	Lignin degradation coefficient	k_LD_		0.528	0.787	0.744	1.037	2.962	–0.058 (^⋆^)
Lignin degradation	Lignin degradation constant	k_1_		0.008	0.005	0.003	0.005	0.003	0.003
	Lignin degradation constant	k_2_		–0.043	–0.053	–0.057	–0.043	–0.037	–0.025
Oxygen uptake	Yield coefficient from oxygen to biomass	*Y*_x/o_	[g X (mmol O_2_)^−1^ ]	0.080	0.018	0.037	0.078	0.009	0.031
	Maintenance coefficient	m_o_	[mmol O_2_ (g X)^−1^ d^−1^]	9.525	20.567	13.046	9.712	19.434	13.526
Carbon dioxide production	Yield of CO_2_ from biomass	*Y*_c/x_	[mmol CO_2_ (g X)^−1^]	20.394	26.741	16.055	10.300	19.901	22.109
	Maintenance coefficient	m_c_	[mmol CO_2_ (g X)^−1^ d^−1^]	6.886	17.091	8.983	5.517	18.094	8.998
Enzyme production	Growth-associated constant	α	[g proteins (g X)^−1^]	0.089	0.154	0.105	0.124	0.126	0.139
	Non growth-associated constant	β	[g proteins (g X)^−1^ d^−1^]	0.011	0.005	0.009	0.009	0.008	0.008

CS, camelina straw; SG, switchgrass; TVm4D, *T. versicolor* m4D; TV52J, *T. versicolor* 52J; PC, *P. chrysosporium*;^⋆^: insignificant estimation.

During the linear log phase (the exponential phase), TV52J showed the highest growing speed (1.210–1.216 d^−1^), followed by TVm4D (0.911–1.005 d^−1^) and PC (0.788–1.005 d^−1^). The self-inhibiting factor, representing the maximum cell concentration that the mycelium can achieve, was ranked highest for TV52J [0.084–0.094 g X (g d.b. substrate)^−1^], followed by those of PC [0.070–0.073 g X (g d.b. substrate)^−1^] and TVm4D [0.047–0.052 g X (g d.b. substrate)^−1^].

The growth ceased after 14-22 d when there was no more room for the cells to develop and divide. During the exponential phase from day 0 to day 14–22 (Figures 3A, B), the hyphae branching occurred aggressively in which the length, volume, and mass of each hypha were increasing and dividing. The microbial biomass was scattered as a network of hyphae on the substrate's surfaces. The stable growth relied upon the polymers from the substrate as a carbon source, which demanded the secretion of specific enzymes (Daniel and Nilsson, 1997; Ordaz et al., 2012; Bari et al., 2016). Those enzymes hydrolyzed the substrate polymers and the soluble hydrolysates diffused through the substrate (Mitchell et al., 2006a; Ruiz-Dueñas and Martínez, 2009; Ordaz et al., 2012; Bari et al., 2016). The diffusion of oxygen from the environment through the immobile gas layer to the fungal cell was stimulated by the oxygen consumption and the substrate's internal oxygen was also transferred to the cell (Mitchell et al., 2006a). At this stage, the space, nutrients, water, and oxygen were readily available for the cell to access given the small fungal biomass concentration, low OUR, and sufficient oxygen restock by the diffusion (Mitchell et al., 2006a). It is possible that the fungal biomass formation entered a nutrient-limiting stage in which the growth solely relied on the nutrient concentration during the early phase of the growth (Xu, 2020). Therefore, the growth happening during this stage was basically biologically limited.

During the latter phase of the growth (the horizontal part of Figures 3A, B), as the hyphal biomass increased, it started to meet those from neighboring microcolonies, and the mycelium started to aggregate and clump (Fuhr et al., 2011; Liu, 2020). The contacts at the tips of the hyphae negatively affected the growth, which is reflected by changes in the direction of growth or a decrease in growth rate (Daniel et al., 1992; Riquelme et al., 1998). Each microorganism has its saturation point beyond which it cannot allow nutrients, air, and water to infiltrate into the core of the aggregate (Moreira et al., 2003; Mitchell et al., 2006a). Only the edges could access oxygen and water and keep dividing until their contact with nutrients from the substrate was terminated (Raghavarao et al., 2003). As the cell concentration increased, other rates related to its growth-associated activities such as OUR, substrate consumption, and heat production, also increased. Oxygen and nutrient concentrations diminished in the adjacent environment of the cell and these reductions were normally faster than the supplying rates toward the fungal cells. Those concentrations continued to decline to a sufficiently low point where the growth rate started to drop (Mitchell et al., 2006a). Furthermore, once the mycelial density was too high, the heat production rate surpassed the heat removal rate regardless of the air-flushing and cooling water outside of the flasks. Toxins as by-products of the growth that could not be released to the environment would be accumulated inside the core. As a result, the high temperature, toxin accumulation, and unavailability of essential substances contributed to the cessation of growth or even death of cells (Mitchell et al., 2006a). Once the cell community approached the carrying capacity of the closed fermentation system, the growth proceeded toward the self-inhibiting stage (Xu, 2020), and the process was restricted by mass transfer (Mitchell et al., 2006a).

The growth kinetics were varied in previous studies depending on the substrate formulation, the SSF conditions, and the fungal strain. The fungal cell development of *T. versicolor* on mixtures of sawdust, coconut fiber, soybean oil, calcium carbonate, coffee husks, and corn bran reached the stationary phase after 30 d of pretreatment time when the substrates were fully colonized, with the maximum fungal biomass concentration of 0.377 g X (g d.b. substrate)^−1^ (Sánchez and Montoya, 2020). *T. versicolor* PSUWC430 and PSUWC431 yielded a maximum specific growth rate of 0.304 and 0.743 d^−1^, respectively, on the same substrate formulations (Montoya et al., 2014, 2015). The maximum specific growth rate of *P. chrysosporium* was 0.411–0.877 d^−1^ and 1.498–1.617 d^−1^ from the growth on cotton stalk under SSF and shallow stationary cultivation, respectively (Shi et al., 2012, 2014), 0.641–0.644 d^−1^ when growing on ground fruit peels (Olorunnisola et al., 2018), and 0.422 d^−1^ from the growth on glucose and ammoniacal nitrogen (Zhongming et al., 2003). Wan (2011) determined that the cell concentration of *C. subvermispora* growing on corn stover increased exponentially between 7-14 d and then started to level off at day 22.

### 3.2. Holocellulose consumption and lignin degradation

Holocellulose, the main carbon and energy source for fungal growth, reduced significantly until 14-22 d, after which the reduction was remarkably slowed as the fungal biomass growth ceased (Figures 3C, D). In CS, holocellulose content was reduced from 58.6 g S (g d.b. substrate)^−1^ in untreated sterile controls to 35.1, 25.7, and 28.6 g S (g d.b. substrate)^−1^ in treatment with TVm4D, TV52J, and PC, respectively, after 14 d, which accounted for 40.1, 56.1, and 51.2%, respectively. During the stationary phase, holocellulose only dropped by 4.5, 8.2, and 4.5% to reach the minimum of 32.4, 20.9, and 25.9 g S (g d.b. substrate)^−1^ at day 30, respectively. Following the same trend with CS, holocellulose of SG decreased from 66.2 g S (g d.b. substrate)^−1^ in untreated sterile controls to 38.4, 33.9, and 36.8 g S (g d.b. substrate)^−1^ in treatment with TVm4D, TV52J, and PC, respectively, after 22, 14 and 18 d, respectively, which were equivalent to 41.9, 48.8, and 44.4%. Longer pretreatment times only decreased the holocellulose by 1.9, 7.7, and 6.3% to reach the lowest of 37.2, 28.8, and 32.6 g S (g d.b. substrate)^−1^ at the end of the time profiles, respectively. The yield coefficient from holocellulose substrate to biomass (*Y*_x/s_) ranked the highest in TV52J (0.249–0.296 g X (g d.b. substrate)^−1^) followed by those of PC (0.213–0.247 g X (g d.b. substrate)^−1^) and TVm4D (0.152-0.180 g X (g d.b. substrate)^−1^). The yield coefficients from the substrate to biomass in this study were similar to those of previous studies. *P. chrysosporium* yielded *Y*_x/s_ of 0.51 g X (g d.b. substrate)^−1^ when growing on glucose and ammoniacal nitrogen (Zhongming et al., 2003), 0.240–0.276 g X (g d.b. substrate)^−1^ on cotton stalk under shallow stationary cultivation (Shi et al., 2012), and 0.437 g X (g d.b. substrate)^−1^ on cotton stalk under SSF (Shi et al., 2014). *Y*_x/s_ was found at 0.577, 0.55, and 0.625 g X (g d.b. substrate)^−1^ in *C. subvermispora, Gibberella fujikuroi*, and *Rhizopus oligosporus*, respectively (Sargantanis et al., 1993; Thibault et al., 2000; Wan, 2011). Higher cell maintenance coefficient (*m*_s_) in TV52J compared to PC and TVm4D indicated that under oxygen enriched environment, TV52J demanded more nutrients and energy than PC and TVm4D to satisfy its metabolisms.

Delignification was highest in TVm4D followed by TV52J and PC. Lignin content decreased from 37.3 g L (g d.b. substrate)^−1^ in untreated sterile CS to the minimum of 23.6, 26.1, and 30.1 g L (g d.b. substrate)^−1^ in CS-TVm4D, CS-TV52J, and CS-PC, which was equivalent to 36.8, 30.1, and 19.3% (Figure 3E). The delignification in SG was 24.4, 14.6, and 10.9% in TVm4D, TV52J, and PC, respectively, which decreased lignin from 26.3 to 19.9, 22.4, and 23.4 g L (g d.b. substrate)^−1^, respectively (Figure 3F). Wan (2011) found that the delignification rate was not constant, instead, it gradually enhanced along with the pretreatment time. Delignification was found to occur even when the fungal growth approached the stationary phase suggesting that complete colonization did not reduce the lignin degradation since the enzymes oxidizing it were still being generated in significant amounts (Sánchez and Montoya, 2020). The inconstant decaying coefficient was observed in the lignocellulosic biomass substrate because of its heterogeneity, therefore, using an exponential equation to illustrate the varying delignification coefficient was appropriate. The negative values of *k*_2_ explained the increasing rate of delignification over time monitored by the higher slope of the lignin degradation curves during the latter stage of the pretreatment. TVm4D showed the highest capability in holocellulose preservation followed by PC and TV52J which agreed well with previous observations. Canam et al. (2011) observed a vigorous growth of TV52J on canola straw in comparison with TVm4D indicated by the remarkable difference in ergosterol content after 84 d (12 weeks). *T. versicolor* m4D was able to colonize and grow on canola straw over 84 d but the growth appeared to slow down after 42 d (Canam et al., 2011). This is likely due to the absence of CDH activity in the mutant strain, which limited its ability to access the cellulosic substrate (Dumonceaux et al., 2001). CDH, an extracellular reductive enzyme, acts as an oxidoreductase to oxidize cellobiose and reduce a variety of substrates, producing organic radicals that can degrade lignin or cellulose, although its activity has been shown primarily to be related to cellulose catabolism (Phillips et al., 2011). The mutant strain of *T. versicolor*, TVm4D, which lacks cellobiose dehydrogenase (CDH), is therefore deficient in accessing and utilizing crystalline cellulose as a carbon source despite having an undamaged complement of hydrolytic cellulases, yet retains the lignin-degrading capabilities of the wild-type parent strain (Canam et al., 2011; Ramirez-Bribiesca et al., 2011). *T. versicolor* was found to degrade more cellulose than hemicellulose in rice straw in comparison with *P. chrysosporium* (Taniguchi et al., 2005). After 24 d of pretreatment, the cellulose content of rice straw reduced from 38.4% to 19.6% with *P. chrysosporium* and 15.8% with *T. versicolor*, while hemicellulose decreased from 24.7% to 13.8% and 18.6%, respectively (Taniguchi et al., 2005). *T. versicolor* also degraded more lignin in rice straw than *P. chrysosporium* did. Lignin content reduced from 25.2% in untreated rice straw to 20.3% in rice straw treated by *P. chrysosporium* and 17.2% in rice straw treated by *T. versicolor* for 24 d (Taniguchi et al., 2005).

### 3.3. Oxygen consumption and carbon dioxide production

**Figure 5:**
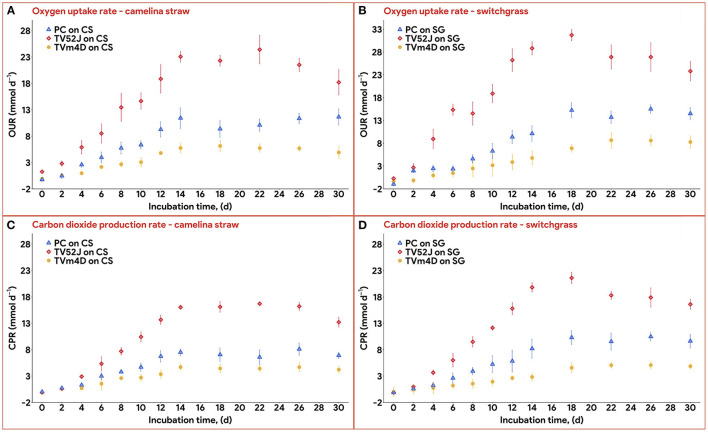
Oxygen uptake rate and carbon dioxide production rate: **(A)**: OUR profiles of the three fungi on CS, **(B)**: OUR profiles of the three fungi on SG, **(C)**: CPR profiles of the three fungi on CS, **(D)**: CPR profile of the three fungi on SG. OUR, oxygen uptake rate; CPR, carbon dioxide production rate; PC, *P. chrysosporium*; TV52J, *T. versicolor* 52J; TVm4D, *T. versicolor* m4D; CS, camelina straw; SG, switchgrass.

**Figure 6:**
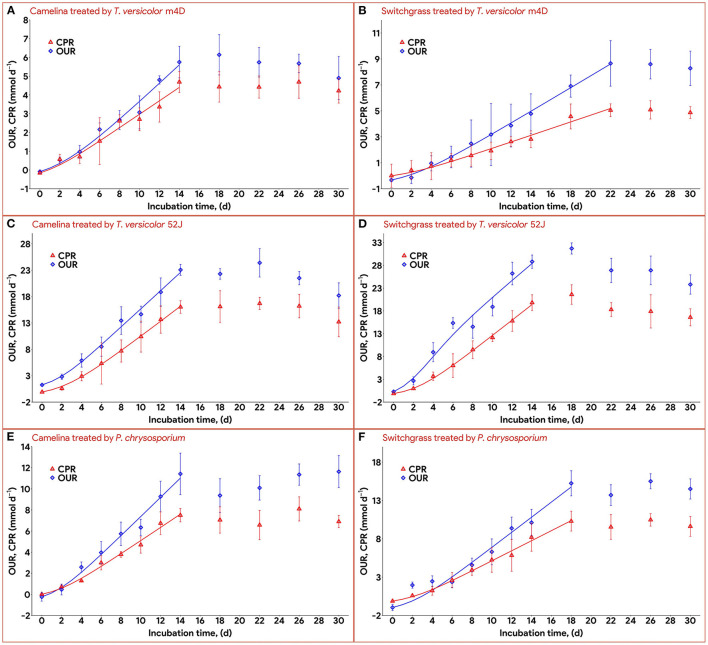
Time profiles of oxygen uptake rate and carbon dioxide production rate during 30 d of microbial pretreatment: **(A)**: profiles of TVm4D on CS, **(B)**: profiles of TVm4D on SG, **(C)**: profiles of TV52J on CS, **(D)**: profiles of TV52J on SG, **(E)**: profiles of PC on CS, **(F)**: profiles of PC on SG. OUR, oxygen uptake rate; CPR, carbon dioxide production rate.

**Figure 7:**
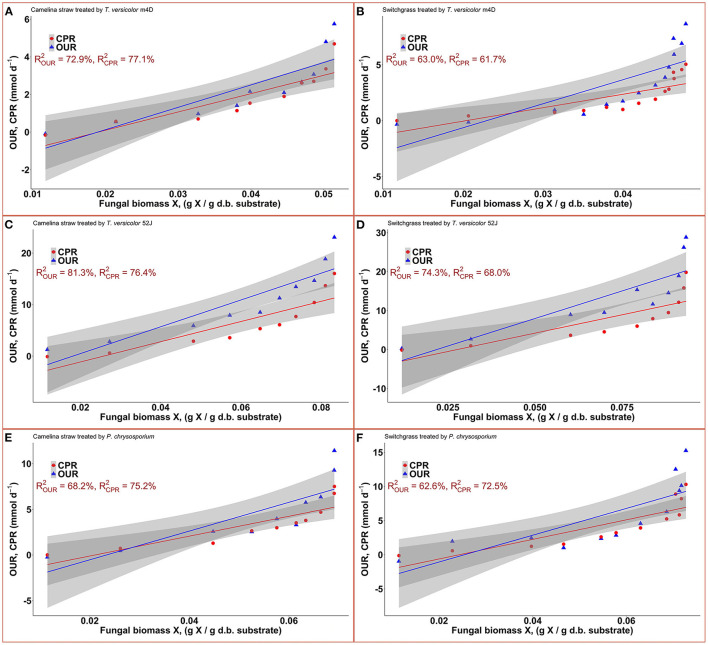
Correlation between fungal biomass content and OUR, CPR: **(A)**: TVm4D on CS, **(B)**: TVm4D on SG, **(C)**: TV52J on CS, **(D)**: TV52J on SG, **(E)**: PC on CS, **(F)**: PC on SG. OUR, oxygen uptake rate; CPR, carbon dioxide production rate.

Data on OUR and CPR is presented in Figure 5. Those two response variables were of interest since they provided the most convenient method to online monitor the growth of a filamentous fungus inside a bioreactor (Mitchell and Krieger, 2006b). It is also proportional to the heat production rate, therefore, providing a useful method for the temperature management of a SSF system (Mitchell and Krieger, 2006b). During the exponential phase of growth, OUR and CPR increased vigorously along with an increase in fungal biomass concentration. As the substrate reached its carrying capacity and fungal growth stagnated, the OUR and CPR remained constant and slightly dropped during the latter stage of the pretreatment. TV52J consumed the highest amount of oxygen and released the greatest amount of carbon dioxide per day followed by PC and TVm4D. This correlated with the higher fungal biomass concentration found in TV52J than that of PC and TVm4D. Only OUR and CPR monitored during the linear log phase of growth were used to fit with models in Equations (6), (7), and illustrated in Figure 6. The yield coefficient from oxygen to the fungal cell was inversely proportional to OUR and was the smallest in TV52J at 0.009–0.018 g X (mmol O_2_)^−1^ followed by those of PC (0.031–0.037 g X (mmol O_2_)^−1^) and TVm4D (0.078–0.080 g X (mmol O_2_)^−1^). In contrast, the maintenance coefficient (*m*_o_) is proportional to OUR and valued highest at 19.434–20.567 mmol O_2_ (g X)^−1^ d^−1^ in TV52J followed by those of PC (13.046–13.526 mmol O_2_ (g X)^−1^ d^−1^) and TVm4D [9.525–9.712 mmol O_2_ (g X)^−1^ d^−1^]. The high amount of oxygen consumption was associated with the high value of the maintenance coefficient from oxygen to fungal biomass indicating the vigorous metabolisms of fungi during the SSF. In previous studies, the yield coefficient from oxygen to the fungal biomass was 0.0061–0.0088 g X (mmol O_2_)^−1^ in *P. chrysosporium* growth on cotton stalk (Shi et al., 2012), 0.0055 g X (mmol O_2_)^−1^ in *C. subvermispora* growth on corn stover (Wan, 2011), 0.015 g X (mmol O_2_)^−1^ in *G. fujikuroi* growth on starch and urea (Pajan et al., 1997). The corresponding maintenance coefficient from oxygen to fungal biomass was 7.469–47.813 g X (mmol O_2_)^−1^ (Shi et al., 2012), 21.8867 g X (mmol O_2_)^−1^ (Wan, 2011), and 19.5 g X (mmol O_2_)^−1^ (Pajan et al., 1997), respectively. The ratio of carbon dioxide production to oxygen consumption (CO_2_/O_2_) was less than one in all substrate-fungus combinations most of the time (Figure 6) representing the simultaneous lignin oxidation-holocellulose metabolisms (Shi et al., 2014). The smaller the CO_2_/O_2_, the stronger the delignification activities (Shi et al., 2014). Therefore, an oxygen-enriched environment is strongly recommended for effective delignification. The plots of OUR and CPR against the fungal biomass concentration X are illustrated in Figure 7. Oxygen uptake rate could be linearly correlated to fungal biomass (X) with determination coefficient of 63.0–72.9% for TVm4D (Figures 7A, B), 74.3–81.3% for TV52J (Figures 7C, D), and 62.6–68.2% for PC (Figures 7E, F). The same linear correlations were also observed in CPR with the determination coefficient of 61.7–77.1% for TVm4D (Figures 7A, B), 68.0–76.4% for TV52J (Figures 7C, D), and 72.5–75.2% for PC (Figures 7E, F). However, the linear correlation might not represent the relationship of OUR and CPR with fungal biomass (X) very well since the data slope tended to increase along with the increase of X. Shi et al. (2012) suggested that a non-linear relationship may occur between OUR and cell concentration X. Therefore, it was necessary to explore more appropriate non-linear models to better predict fungal biomass from instantaneous and inexpensive measurements including OUR and CPR.

### 3.4. Enzyme formation

**Figure 8:**
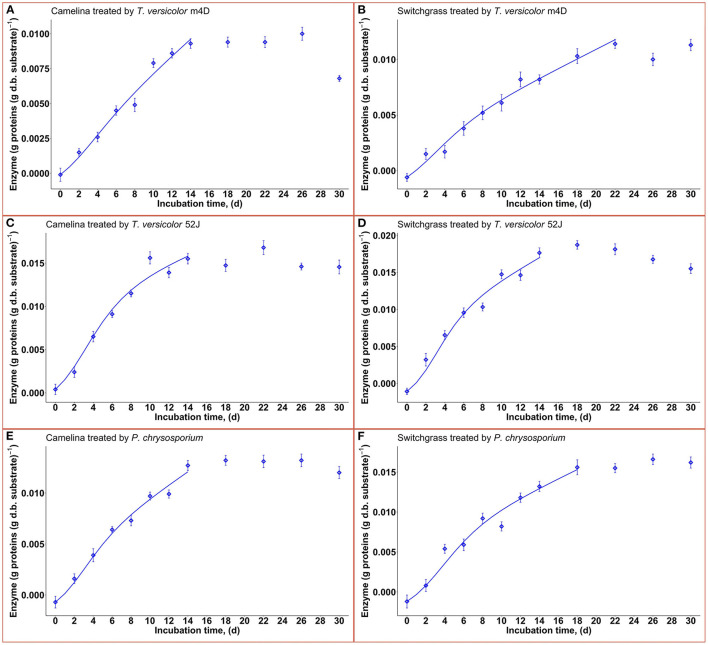
Time profiles of enzyme production rate during 30 d of microbial pretreatment: **(A)**: profiles of TVm4D on CS, **(B)**: profiles of TVm4D on SG, **(C)**: profiles of TV52J on CS, **(D)**: profiles of TV52J on SG, **(E)**: profiles of PC on CS, **(F)**: Profiles of PC on SG; The continous lines were determined by the proposed models.

Enzyme production was examined to give a broader view of product formation by fungus vs. cell growth since it was not the main interest of this study regarding the microbial pretreatment of lignocellulosic substrate for biofuel production. Following the same trend with fungal growth (X), OUR, and CPR, the enzyme production rate increased during the linear log phase of growth and stagnated after that (Figure 8). The higher values of α than that of β suggested that enzyme production might be growth-associated. The growth-associated constant (α) ranked highest in TV52J [0.126–0.154 g proteins (g X)^−1^] followed by that of PC (0.105–0.139 g proteins (g X)^−1^) and TVm4D [0.089–0.124 g proteins (g X)^−1^]. Cellulolytic and ligninolytic enzyme productions might be correlated to fungal biomass growth and substrate metabolisms (Shi et al., 2012). Shi et al. (2012) determined that ligninolytic enzyme production of PC growth on cotton stalk was not directly influenced by fungal growth since α and β were on the same magnitude. However, their models failed to estimate cellulase production. The enzyme production model proposed by Wan (2011) also generated a poor prediction because of the complicated enzyme systems during the SSF of *C. subvermispora* on corn stover. Enzyme generation was observed to be independent of metabolic energy provided by carbohydrates (Gaden, 1959), and the energy demand of PC fungus was higher than that provided by delignification (Shi et al., 2012). An overall trend of decline in enzyme production after a specific incubation time was also observed in *T. versicolor, P. ostreatus*, and *L. edodes* (Montoya et al., 2015). It was strongly recommended to monitor individual enzymes as well as provide more sophisticated models to predict each one of them.

### 3.5. Model diagnostics

The diagnostics of the nonlinear models were conducted based on 4 hypotheses including: 1) the correct mean function; 2) the correctness of the assumption of homogeneity of variances (homoscedasticity); 3) the errors of measurements were normally distributed; and 4) the errors of measurements were mutually independent (Ritz and Streibig, 2008). The normality and independence of measurement errors were reliably determined by the replication of treatments, the randomization of sampling, and the repeated final measurements (the repeatability of the analysis). In general, if the model explains the mean structure of the dataset suitably, then the predicted regression curve should follow the trend of the data. Therefore, the model could be examined visually by plotting the regression curves against the observations. The suitability of the fitness was also inspected by evaluating whether the measured data were allocated randomly (non-systematically) around the fitted curve by generating a residual plot.

With the estimated parameters indicated in Table 1, the predicted values of fungal biomass (X), holocellulose (S), and lignin content (L) were plotted against the experimental observations (Figure 4). The calculated results by the proposed hybrid logistic-Monod model agreed well with the batch culture experimental data, which was indicated by the high correlation coefficients and the random distribution of the residuals around zero (see *model's normality check* in Supplementary Figures 9A–C, 18A–C, 27A–C, 36A–C, 45A–C, 54A–C). The insignificance of the estimated *k*_LD_ in the case of SG-PC may have resulted from the insignificant reduction of lignin content in SG by *P. chrysosporium*. The predictions of OUR, CPR, and enzyme formation during the linear log phase of growth are shown in Figures 6, 8, respectively, and the regression curves also agreed well with the observations. The residuals of OUR, CPR, and enzyme models were also randomly distributed around the horizontal zero line without showing any obvious trend or pattern indicating the correctness of the models and the assumptions (see *model's normality check* in Supplementary Figures 9D–F, 18D–F, 27D–F, 36D–F, 45D–F, 54D–F). In addition, the standard deviation of the errors of prediction (the standard error of the estimate, σ_est_) was also calculated and indicated in Supplementary Figures 9, 18, 27, 36, 45, 54. The very small values of σ_est_ represent the high accuracy of the predictions.

### 3.6. Discussion on the model applications in the context of biomass-based biorefineries

Simultaneous experimental and modeling design is recommended to develop the production scale SSF bioreactor (Mitchell et al., 2006b). Supplementary Figure 55 illustrates the strategy of using models to design and optimize the operation of SSF bioreactors. Determination of bioreactor type, modeling the growth kinetics and testing it on a laboratory scale is the very first steps of the process. In the context of biorefineries, the physiochemical quality of pellets (i.e., durability and heating value) or the structural characteristics of the substrate (i.e., the content of carbohydrates or enzymatic digestibility) are the targeted product information. After optimizing the product quality by a particular microorganism on a specific lignocellulosic substrate under lab scale, engineers and scientists will have enough data to develop large-scale process. After the bioreactor is built with the assistance of the model, data obtained from the operation can be used to enhance the model, and hence making the model more useful. The performance of the bioreactor can be tuned and significantly enhanced by enforcing the control strategy; in this case, the model is also useful in establishing those control schemes. In general, a mathematical model of an SSF bioreactor demands two sub-models including 1) the growth kinetics sub-model which represents the biological aspects of the microorganism and 2) the mass and energy balances and transport circumstance sub-model (Mitchell and Krieger, 2006a). The first model, which was developed in this study, provides essential information to develop the second model (Supplementary Figure 56). The fungal biomass equation can be used to predict the metabolic heat generation and hence assist the cooling system design. Or the headspace gas equations can be used to predict the fungal biomass content, which is cumbersome to measure, and therefore provide a quick method to determine the growth condition. They can also be used to calculate the amount of oxygen/air to be supplied to the bioreactor. The holocellulose and lignin equations can be used to forecast the product's targets and then adjust the operating condition of the reactor. It is highly recommended that the second sub-model is developed to generate data for both parts of the model.

## 4. Conclusions

In this study, a series of mathematical models was proposed to characterize the growth of *T. versicolor* 52J, *T. versicolor* m4D, and *P. chrysosporium* on camelina straw and switchgrass for 30 d. Fungal cell concentration was described well by a hybrid logistic-Monod equation along with holocellulosic substrate consumption and lignin degradation. The oxygen consumption and carbon dioxide generation were also modeled to prepare a prompt method for fungal biomass prediction as well as evaluating the greenhouse gas emission. The enzyme production was modeled as an attempt to correlate the fungal biomass with the product formation. The hybrid equation along with the substrate consumption and lignin degradation equations explained the growth kinetics very well. It was observed that:

- The growth of TV52J, TVm4D, and PC during 30 d of pretreatment included two phases: 1) the linear log phase (exponential phase) happened from day 0 to day 14 or 22 in which the growth was basically biologically limited, and 2) the stagnation phase occurred from day 14 or 22 to day 30 in which the growth was restricted by mass transfer.
- During the linear log phase, TV52J gave the highest maximum specific growth rate and maximum cell concentration while its mutant strain TVm4D performed best in terms of holocellulose preservation and delignification.
- TV52J performed the highest OUR and CPR followed by PC and TVm4D. The OUR and CPR were directly correlated to the fungal biomass concentration; however, a more sophisticated non-linear relationship might explain those correlations better than linearity.
- The simultaneous lignin oxidation-holocellulose metabolisms were observed in all fungi-substrate combinations.
- Although the enzyme model agreed well with the data, in future work we will monitor individual enzymes to integrate them within a more sophisticated model.
- The study provided information on one of the two important sub-models of SSF bioreactors: the biological parameters of the microbial growth kinetics sub-model. It is necessary to examine the balance/transport sub-model for the modeling of heat and mass transfer and the SSF bioreactor's energy balance to estimate the system's thermodynamics parameters.
- The application of mechatronics systems for online monitoring and controlling the SSF processes is highly recommended to study large-scale bioreactors.

## Data availability statement

The original contributions presented in the study are included in the article/Supplementary material, further inquiries can be directed to the corresponding authors.

## Author contributions

CD: conceptualization, methodology, software, validation, formal analysis, investigation, data curation, writing—original draft, and visualization. LT, EM, and TD: writing—review and editing, supervision, project administration, and funding acquisition. All authors contributed to the article and approved the submitted version.
