# Supplementary material for: Modeling the microbial pretreatment of camelina straw and switchgrass by Trametes versicolor and Phanerochaete chrysosporium via solid-state fermentation process: A growth kinetic sub-model in the context of biomass-based biorefineries
Source: Front Microbiol. 2023 Apr 6;14:1130196. doi: 10.3389/fmicb.2023.1130196 (PMC10117130; doi:10.3389/fmicb.2023.1130196)
Supplement: Supplementary file 1 [file Data_Sheet_1.PDF]

Supplementary

Table S1: The advantages and disadvantages of biological pretreatment in comparison with other pretreatment methods.

Pretreatment	Advantages	Disadvantages	Source
		**Biological Pretreatment**	
Ligninolytic Abilities of Fungi & Enzymatic Pretreatment	1) Low energy consumption due to lower temperatures and pressures of pretreatment. 2) Environmentally friendly due to no harsh chemicals requirements. 3) Selectivity of delignification without cellulose consumption results in higher-quality products and lower processing costs. 4) Lower waste and environmental impacts due to reusable and recycled microorganisms.	1) Time-consuming which might increase the cost of pretreatment. 2) Cost: Some biological pretreatment methods can be expensive due to the demand for specialized equipment, specific microorganisms, or enzymes. 3) Limited effectiveness due to the incomplete breakdown of lignocellulosic materials, resulting in lower fermentable sugar yields and therefore reducing the efficiency of downstream processes. 4) Sensitivity to environmental conditions such as temperature, moisture content, pH, etc. 5) Risk of contamination due to undesired microorganisms, which can compete with or inhibit the desired microorganisms, reducing the effectiveness of the pretreatment. 6) Scale-up difficulties due to the need for strict environmental conditions and the sensitivity of the microorganisms to changes in these conditions.	Sharma et al. (2019); Su et al. (2018); Wan and Li (2012)
		**Physical pretreatment**	

Mechanical Comminution	1) A mature process. 2) Increased surface area makes it easier for enzymes to access and break down the cellulose and hemicellulose components of the materials. 3) Smaller particle size reduces the resistance to further processing, such as hydrolysis or fermentation leading to increased yields and reduced processing times. 4) Improved uniformity which can result in more consistent processing conditions and higher product quality. 5) Reduced energy requirements of subsequent processing steps. 6) Access to previously inaccessible materials.	1) High energy requirement thus high cost of pretreatment. 2) Production of fines. 3) Heat generation which can damage the lignocellulose and reduce the effectiveness of subsequent pretreatment or conversion processes. 4) Incomplete delignification. 5) High equipment costs.	Zhang et al. (2010)
Drying and Concentration	1) Increased storage stability. 2) Improved handling properties. 3) Reduced transportation costs. 4) Reduced energy requirements for subsequent processing steps due to less energy being required to remove moisture. 5) Improved conversion efficiency during subsequent chemical or enzymatic treatments due to the absence of water.	1) High energy requirement particularly if the material is wet and needs to be dried to a low moisture content. 2) Reduced yield due to material loss. 3) Thermal degradation. 4) Increased storage and handling requirements such as protective packaging or temperature-controlled storage, to prevent degradation or spoilage. 5) Risk of contamination.	Show et al. (2019); Verma et al. (2017)
Granulometric Separation	1) Improved process efficiency due to the easy treatment of different fractions. 2) Reduced energy requirements due to lower energy required to convert smaller particles. 3) Improved product quality as different size fractions may have different properties that are desirable for specific applications. 4) Reduced processing cost by allowing for more efficient use of downstream processing equipment, and reducing the amount of material that needs to be processed. 5) Increased production yield by improving the accessibility of the material to subsequent processing steps, and by reducing losses due to inefficient processing.	1) Energy-intensive process. 2) Loss of valuable components. 3) Widely varied particle size distribution which can affect the effectiveness of subsequent processing steps. 4) Expensive equipment requirements. 5) Negative environmental impacts due to waste.	Montero et al. (2014)
High-Energy Radiation (Microwave Oven)	1) Faster processing time which can save energy and reduce operational costs, as well as increase the overall productivity of the process. 2) Reduced energy consumption as compared to steam explosion or acid hydrolysis, which can result in lower operational costs and make the process more environmentally sustainable. 3) Improved product yield. 4) Enhanced digestibility. 5) Increased selectivity.	1) Uneven heating which can lead to non-uniform processing and product quality. 2) Specialized equipment requirement which can be expensive to acquire and maintain. 3) Sensitivity to biomass characteristics. 4) Safety concerns. 5) Limited penetration.	Kostas et al. (2017); Li et al. (2016)

High-Energy Radiation (Electron Beam Radiation)	1) Efficient and fast process which can save time and energy in the subsequent processing steps. 2) Enhanced product yield. 3) Selectivity which can preserve cellulose content and reduce the formation of unwanted byproducts, which can reduce the environmental impact of the process. 4) Reduced environmental impact due to the lack of harmful chemicals. 5) Improved digestibility.	1) Specialized equipment requirement which can be expensive to acquire and maintain. 2) High-energy requirements which can result in high energy costs and make it difficult to implement the process on a large scale. 3) Limited penetration which can result in incomplete processing and non-uniform product quality. 4) Safety concerns due to hazardous radiation. 5) Potential changes to material properties.	Karthika et al. (2012); Sung and Shin (2011)
Pyrolysis	1) Efficient and fast process which can save time and energy in the subsequent processing steps. 2) Versatility due to its suitability with a wide range of materials. 3) Reduced environmental impacts due to the lack of harmful chemicals and the usefulness of its byproducts. 4) Improved digestibility. 5) Selectivity.	1) High energy requirements to heat the biomass to the required temperatures, which can result in high energy costs and make it difficult to implement the process on a large scale. 2) Variability in the process due to the highly variable optimal conditions regarding the type and characteristics of the biomass being processed. 3) Byproduct management which can be challenging to manage and may require additional processing steps to be useful or safe for disposal. 4) Reduced product yield due to mass loss. 5) Emissions and safety concerns regarding volatile organic compounds and greenhouse gases and high temperatures.	Kan et al. (2016); Xiao et al. (2010)
		**Chemical pretreatment**	
Acid	1) A mature process. 2) Increased digestibility. 3) Reduced lignin content. 4) Improved biomass properties. 5) Inexpensive due to widely available chemicals and non-complex equipment. 6) Increased product yield.	1) Corrosion and safety concerns due to strong acids. 2) High energy requirements due to high temperatures required to heat the acid. 3) Acid recovery and disposal which can be costly and time-consuming and might pollute the environment. 4) Formation of inhibitory compounds such as furfural and hydroxymethylfurfural, which can interfere with subsequent fermentation processes. Additional processing steps may be required to remove these compounds, which can add to the overall cost of the process. 5) Loss of cellulose.	Mafe et al. (2015); Kim and Lee (2002)

Alkaline	1) Increased digestibility by dissolving hemicellulose and partially removing lignin, which can increase the accessibility of the remaining cellulose for subsequent enzymatic hydrolysis. 2) Mild reaction conditions as compared to other pretreatment methods, which can reduce the energy requirements and operating costs of the process. 3) Reduced formation of inhibitory compounds. 4) Increased yields of valuable co-products such as lignin and hemicellulose derivatives. These co-products can be used as feedstocks for the production of a range of high-value chemicals and materials, which can increase the overall economic viability of the process. 5) Environmentally friendly due to the use of non-toxic and environmentally friendly chemicals, such as sodium hydroxide.	1) Reduced lignin content makes the material more susceptible to degradation and erosion. 2) Alkali recovery and disposal which can be costly and time-consuming and might pollute the environment. 3) Loss of hemicellulose. 4) Potential formation of toxic compounds such as chlorinated organic compounds, which can be harmful to the environment and human health if not properly managed. Additional processing steps may be required to remove these compounds, which can add to the overall cost of the process. 5) Limited applicability depending on the biomass type and process conditions.	Kim et al. (2016)
Ionic Liquid (IL)	1) Efficient biomass fractionation. 2) High selectivity which can remove lignin while leaving the cellulose and hemicellulose intact. 3) Low environmental impact due to the non-volatile and low toxic characteristics of ionic liquid. 4) Reduced energy consumption since the pretreatment can be carried out at lower temperatures and with less energy compared to traditional pretreatment methods, such as steam explosion or acid hydrolysis. 5) Versatility since Ionic liquids can be tailored to different biomass feedstocks and desired applications.	1) High cost since Ionic liquids can be expensive to produce. 2) Difficulty in recovering ionic liquids because ionic liquids are non-volatile and recovering them from the biomass can be challenging and may require additional separation steps, which can increase the overall cost of the process. 3) Potential environmental impacts due to their (low) toxicity. 4) Limited compatibility with some enzymes because some ionic liquids can inhibit the activity of enzymes used in downstream processes, which can reduce the efficiency of the conversion process. 5) Limited scalability due to challenges in efficient mixing, heat transfer, and the need for specialized equipment.	Zhang et al. (2017)

Organosolv	1) High lignin removal rate. 2) Production of high-quality lignin because the lignin that is removed during organosolv pretreatment is typical of high quality and can be used for high-value applications, such as the production of bio-based chemicals or materials. 3) Reduced environmental impacts due to the use of organic solvents, such as ethanol or acetone, which are less toxic and more environmentally friendly than traditional pretreatment methods that use harsh chemicals. 4) Recovery of valuable byproducts since it produces a lignin-rich liquid fraction and a cellulose-rich solid fraction. 5) High selectivity since it can remove lignin from lignocellulosic materials while leaving the cellulose and hemicellulose intact.	1) High cost due to the cost of the organic solvents used. 2) High energy demand due to the use of high temperatures and pressures. 3) Recovery of the organic solvent may require additional energy input, which can increase the overall cost of the process. 4) Limited scalability due to the high energy requirements, difficulties in solvent recovery and recycling, and the need for specialized equipment. 5) Limited compatibility with some enzymes because the organic solvents used in organosolv pretreatment can have negative effects on the activity of enzymes used in downstream processes, which can reduce the overall efficiency of the conversion process. 6) Byproduct contamination since the lignin-rich liquid fraction produced by organosolv pretreatment may contain some residual sugars and inhibitors that can negatively impact downstream fermentation processes.	Borand and Karaosmanoğlu (2018); Zhou et al. (2018)
Ozonolysis	1) Increased digestibility. 2) Reduced toxicity since Ozonolysis can reduce the concentration of some toxic compounds such as furans and phenols, making the material safer to handle and process. 3) Improved bleachability since the removal of lignin by ozonolysis can improve the ability of lignocellulosic materials to be bleached, which can be important for paper production. 4) Reduced environmental impacts since Ozonolysis is a relatively environmentally friendly pretreatment method, as it does not produce toxic byproducts and does not require high temperatures or chemicals.	1) High cost. 2) Production of ozone-depleting compounds such as peroxides and aldehydes, which can have negative environmental impacts. 3) Lower cellulose content since Ozonolysis can break down not only the lignin and hemicellulose but also some of the cellulose in the lignocellulosic material. 4) Limited effectiveness for some materials since Ozonolysis may not be effective for some types of lignocellulosic materials, depending on their composition and structure. 5) Occupational health and safety concerns since Ozone gas can be hazardous to human health at high concentrations, and proper safety measures and equipment must be used to prevent exposure to workers.	Travaini et al. (2016)
Oxidative Delignification	1) Increased digestibility. 2) Increased cellulose proportion. 3) Improved product quality. 4) Reduced environmental impacts since it produces fewer toxic byproducts. 5) Reduced energy requirements since it can be performed at lower temperatures and pressures than other pretreatment methods.	1) High cost. 2) Production of toxic byproducts such as lignin-derived phenols, which can have negative environmental impacts. 3) Decreased material strength. 4) Limited effectiveness for some materials. 5) Safety concerns.	Zhou et al. (2022)

Sulfite	1) Increased digestibility. 2) Increased cellulose proportion. 3) Improved product quality. 4) Reduced environmental impacts as it produces fewer toxic byproducts compared to some other pretreatment methods. 5) Reduced energy requirements since it can be performed at lower temperatures and pressures than some other pretreatment methods. 6) Versatility since it can be effective for a variety of lignocellulosic materials, including hardwoods, softwoods, and agricultural residues.	1) High cost. 2) Limited effectiveness for some materials. 3) Generation of sulfite waste since it generates sulfite waste, which can have negative environmental impacts if not properly managed. 4) Formation of inhibitory compounds such as furfural and hydroxymethylfurfural, which can negatively impact subsequent processes, such as fermentation. 5) Safety concerns since the use of chemicals in sulfite pretreatment can pose occupational health and safety risks, and appropriate safety measures and equipment must be used to prevent exposure to workers.	Huang et al. (2022); Wang et al. (2009)
	**Physicochemical Pretreatment**		
Steam Explosion	1) Increased accessibility of enzymes. 2) Reduced inhibitor formation since during steam explosion, some of the hemicellulose and lignin components are broken down into less inhibitory compounds, reducing the formation of inhibitors that can interfere with downstream fermentation processes. 3) Reduced energy and chemical requirements since it is a relatively mild pretreatment process that does not require the use of harsh chemicals, such as acids or bases, which are required for other pretreatment methods. This can reduce the overall energy and chemical requirements for bioconversion processes. 4) Potential for byproduct generation such as lignin, which can be used for other purposes, such as energy generation. 5) Reduced capital costs since it requires relatively simple equipment and has lower capital costs, compared to other pretreatment methods.	1) High energy consumption since it requires a significant amount of energy to generate the high-pressure steam necessary to rupture the lignocellulosic material's cell walls. This can make the process expensive, both in terms of energy costs and equipment requirements. 2) Incomplete delignification because it may not completely remove lignin from the cellulose. 3) Formation of inhibitory compounds: Although steam explosion can reduce the formation of some inhibitory compounds, it can also generate others, such as furfural and hydroxymethylfurfural (HMF), which can reduce the efficiency of downstream fermentation processes. 4) Environmental impacts due to high energy consumption. 5) Variability in biomass response: Different lignocellulosic materials may respond differently to steam explosion pretreatment, making process optimization difficult and leading to inconsistent yields and product quality.	Auxenfans et al. (2017); Duque et al. (2016)

Liquid Hot Water (LHW)	1) Mild process conditions since it does not require the use of harsh chemicals or high-energy inputs. This can lead to lower costs and reduced environmental impact. 2) High sugar yields. 3) Reduced inhibitor formation: LHW pretreatment can result in reduced formation of inhibitors such as furfural and HMF, which can interfere with downstream fermentation processes. 4) Retention of lignin: LHW pretreatment can retain some of the lignin of the biomass, which can act as a natural barrier against microbial attack and improve the structural stability of the resulting hydrolyzate. 5) Potential for byproduct generation such as acetic acid, which can be used for other purposes, such as energy generation. 6) Improved product quality: LHW pretreatment can result in a more uniform biomass particle size, which can improve product quality and reduce the likelihood of equipment blockages during downstream processing.	1) Equipment requirements since it requires high-pressure vessels to generate and maintain the high temperature and pressure conditions necessary for the process. This can be costly, and the equipment may require regular maintenance. 2) Limited delignification since it may not completely remove lignin from the biomass, leading to lower sugar yields during subsequent enzymatic hydrolysis. 3) Formation of degradation products such as organic acids, that can lower the pH of the hydrolyzate and interfere with downstream fermentation processes. 4) Variability in biomass response. 5) Energy requirement since it still requires a significant amount of energy to heat the water to the required temperature and pressure. This can make the process expensive and environmentally costly. 6) Environmental impacts because it can result in the release of organic compounds and greenhouse gases, contributing to environmental pollution and climate change.	Chen et al. (2022)
Ammonia Fiber Expansion (AFEX)	1) Reduced energy requirements since it does not require the use of high-pressure vessels, and it operates at atmospheric pressure, resulting in lower energy requirements compared to other pretreatment methods. 2) High sugar yields. 3) Reduced inhibitor formation. 4) Preservation of lignin. 5) Minimal generation of wastewater. 6) Versatility.	1) Ammonia toxicity: Ammonia can be toxic to microorganisms, which may affect subsequent fermentation processes. This can require additional steps to remove residual ammonia or to select microorganisms that are more resistant to ammonia toxicity. 2) High ammonia requirements: AFEX pretreatment requires a large amount of ammonia, which can make the process expensive. 3) Limited delignification. 4) Reduced cellulose digestibility: While AFEX pretreatment can improve cellulose accessibility to enzymes, it can also lead to a reduction in cellulose digestibility due to the formation of ammonia-pretreated lignocellulosic residues (APLRs) during the process. 5) Limited scalability: AFEX pretreatment may not be scalable to larger production volumes due to the high ammonia requirements and the need for large, specialized equipment. 6) Safety concerns: Ammonia is a hazardous material, requiring careful handling and storage to prevent accidents or environmental damage.	Chundawat et al. (2020); Lau et al. (2009)

Ammonia Recycled Percolation (ARP)	1) Cost-effective. 2) High efficiency. 3) Low chemical usage since the amount of ammonia used in ARP pretreatment is much lower than in other ammonia-based pretreatment methods, making it more environmentally friendly and safer for operators. 3) Low waste production since the ammonia used in ARP pretreatment is recycled, reducing the production of waste and making it a more sustainable method compared to other pretreatment techniques. 4) Improved enzymatic hydrolysis. 5) Better product quality by reducing the amount of inhibitors, such as furfural and hydroxymethylfurfural (HMF), which can negatively affect subsequent bioconversion processes.	1) High energy consumption since it requires a high level of energy input, particularly during the ammonia recovery step, which can increase operating costs and impact the overall environmental sustainability of the process. 2) Limited applicability since it has been found to be more effective for hardwoods and herbaceous biomass, but less effective for softwoods and agricultural residues. 3) Ammonia toxicity since Ammonia can be hazardous to workers if not handled properly. High levels of ammonia in the air can cause respiratory problems, and contact with liquid ammonia can cause chemical burns. Therefore, operators need to follow strict safety protocols and use appropriate protective equipment. 4) Environmental impact since the use of ammonia can still have negative environmental impacts if not properly managed. Ammonia can leach into soil and water systems and contribute to eutrophication and other environmental problems. 5) Risk of biomass degradation due to the high pH and temperature conditions. This can reduce the overall quality of the lignocellulosic material and negatively impact subsequent bioconversion processes.	Zhao et al. (2020); Iyer et al. (1996); Yoon et al. (1995)
Soaking in Aqueous Ammonia (SAA)	1) Efficient removal of lignin. 2) Improved enzymatic hydrolysis. 3) Reduced inhibitor formation. 4) Environmental friendliness. 5) Cost-effective.	1) Ammonia handling due to the use of concentrated aqueous ammonia, which is a hazardous material that requires special handling and safety precautions. 2) Ammonia recovery. 3) High capital costs due to the need for specialized equipment such as pressurized vessels, ammonia storage and recovery systems, and pH control systems. 4) Long pretreatment times range from several hours to several days, depending on the specific conditions used. This can add to the overall cost and time required for the process. 5) Low selectivity. 6) Potential environmental impact due to the release of ammonia or other chemicals used in the process.	Pandey et al. (2019); Kang et al. (2012); Kim et al. (2008)

Cellulose Solvent-Based Lignocellulose (CSL)	1) Highly efficient lignin removal. 2) Low energy consumption since it can be carried out at low temperatures and atmospheric pressure, which reduces the energy consumption and operational costs of the process. 3) Short pretreatment time. 4) High selectivity. 5) Low toxicity. 6) High-quality lignin byproduct.	1) High solvent cost. 2) Solvent recovery. 3) High capital costs due to the need for specialized equipment such as pressurized vessels, solvent storage and recovery systems, and pH control systems. 4) Potential environmental impact from the release of solvents or other chemicals used in the process. 5) Limited scale-up: CSL pretreatment is a relatively new technology, and large-scale implementation may require further research and development. 6) Compatibility issues.	Zhu et al. (2009); Sathitsuksanoh et al. (2013)
Cellulose Solvent- and Organic Solvent-Based Lignocellulose Fractionation (COSLIF)	1) Selective Fractionation. 2) High Yield. 3) Low Energy Consumption. 4) Reduced Environmental Impact. 5) Versatility.	1) Cost: The use of cellulose solvent and organic solvents, which can be expensive, may increase the cost of the process, making it less economically viable for some applications. 2) Safety: The organic solvents used in the process can be hazardous, and precautions must be taken to ensure worker safety and avoid environmental contamination. 3) Process complexity: COSLIF pretreatment involves a series of steps, including solvent extraction, washing, and drying, which can be time-consuming and add to the overall complexity of the process. 4) Yield variability. 5) Residual solvent.	Sathitsuksanoh et al. (2012)
Aqueous N-Methylmorpholine-N-Oxide (NMMO)	1) High Selectivity. 2) Low Energy Consumption. 3) Minimal Environmental Impact: NMMO is a relatively benign solvent that is non-toxic and biodegradable, which makes it a safer and more environmentally friendly option than some of the harsher chemicals used in other pretreatment methods. 4) High Yield. 5) Versatility.	1) Cost. 2) Equipment and Infrastructure. 3) Limited Scale-Up: NMMO pretreatment has been primarily developed and used in lab-scale studies, with limited studies at larger scales. Therefore, scaling up the process to an industrial scale may require further research and development. 4) Disposal of Used Solvent: The disposal of used NMMO solvent may be challenging, as it is a non-volatile, high boiling-point solvent that requires specialized disposal methods. 5) Long Processing Time.	Rosenau and French (2021); Li et al. (2012)

Urea/Sodium Hydroxide	1) Low Cost: Urea and NaOH are relatively inexpensive chemicals, making this pretreatment method cost-effective. 2) High Yield. 3) Selectivity: Urea/NaOH pretreatment is selective for hemicellulose, which can be easily separated from the other components of the lignocellulosic material, such as cellulose and lignin. 4) Reduced Enzyme Requirement: The pretreated material may require lower amounts of enzymes for subsequent hydrolysis due to the high degree of hemicellulose removal. 5) Reduced Inhibitor Formation. 6) Reduced Environmental Impact: Urea/NaOH pretreatment uses relatively benign chemicals that have a low environmental impact, making it an environmentally friendly option for biomass processing.	1) Harsh Conditions. 2) Corrosion. 3) Limited Lignin Removal: Urea/NaOH pretreatment is selective for hemicellulose and has limited lignin removal. This can be a disadvantage if the application requires lignin removal or if the lignin-containing materials cause downstream processing issues. 4) Scaling Up: The scalability of the Urea/NaOH pretreatment process may require further research and development as it is primarily used in lab-scale studies. 5) Environmental Impact.	Jing et al. (2022); Shao et al. (2020)
N, N-Dimethylacetamide (DMac)/LiCl	1) High Yield. 2) Selectivity. 3) Low Toxicity: DMac and LiCl are relatively non-toxic chemicals, making this pretreatment method environmentally friendly. 4) Low Viscosity: The DMac/LiCl solution has low viscosity, which can improve the mass transfer of the solution and make the process more efficient. 5) Reduced Enzyme Requirement. 6) Reduced Environmental Impact.	1) Cost: DMac and LiCl are relatively expensive chemicals. 2) High-Temperature Requirement: DMac/LiCl pretreatment requires high temperatures (typically between 120-160 °C) to achieve effective cellulose fractionation, which can increase energy consumption and costs. 3) Corrosion: DMac/LiCl is corrosive and can lead to the wear and tear of processing equipment. 4) Difficulty in Removing LiCl. 5) Limitations in Lignin Removal. 6) Potential Environmental Impacts.	Ali et al. (2020); Zhang et al. (2014)
		**Combination of pretreatments**	
Alkaline + Dilute Acid (ALK-DA)	1) High lignin Removal. 2) Improved Saccharification. 3) Reduced Enzyme Requirement. 4) Selectivity. 5) Low Environmental Impact. 6) High Yield.	1) High Energy Requirement due to the need for both alkaline and acid hydrolysis steps, which can increase the overall cost of the process. 2) Corrosion. 3) Formation of Inhibitory Compounds such as furfural and 5-hydroxymethylfurfural (HMF), which can inhibit subsequent fermentation steps. 6) Complexity: ALK-DA pretreatment is a complex process that requires careful optimization of both the alkaline and acid hydrolysis steps. 7) Limited Application to Some Feedstocks. 8) Potential Environmental Impacts.	Mood et al. (2013)

Alkaline + Ionic Liquid (ALK-IL)	1) Improved lignin removal. 2) Increased cellulose accessibility. 3) Reduced environmental impact: Ionic liquids are non-volatile and non-flammable, making them safer to use and handle than traditional organic solvents. Furthermore, the ALK-IL pretreatment can be carried out under mild conditions, requiring lower temperatures and shorter reaction times, which results in reduced energy consumption and environmental impact. 4) Enhanced sugar recovery. 5) Versatility.	1) Cost. 2) Compatibility. 3) Ionic liquid recovery. 4) Corrosiveness. 5) Toxicity. 6) High viscosity.	Lethesh et al. (2020); Mood et al. (2013); Nguyen et al. (2010)
Dilute Acid + Steam Explosion (DA-SExp.)	1) Enhanced cellulose accessibility. 2) Reduced enzyme loading. 3) Improved hemicellulose recovery. 4) Cost-effectiveness. 5) Reduced environmental impact. 6) Compatibility.	1) Corrosiveness. 2) Sugar degradation. 3) Sugar loss. 4) Inhibitory compounds. 5) Acid neutralization. 6) Waste disposal.	McIntosh et al. (2016); Chen et al. (2011); Emmel et al. (2003)
Supercritical CO_2 + Steam Explosion (SCCCO-SExp.)	1) Enhanced enzymatic hydrolysis. 2) Reduced formation of inhibitory compounds. 3) Reduced energy requirements. 4) Reduced environmental impact. 5) Possibility for hemicellulose recovery. 6) Improved sugar yields	1) High capital costs: The use of supercritical CO_2 requires expensive equipment, and the capital cost of the SCCCO-SExp. process can be higher than some other pretreatment methods. 2) Complexity. CO_2 utilization. 3) High-pressure safety. 4) Equipment corrosion. 5) Feedstock composition.	Alinia et al. (2010)
Organosolv + Biological (Bio-Organosolv)	1) High-quality lignin. 2) Low chemical usage: Compared to traditional Organosolv pretreatment, Bio-Organosolv requires lower chemical usage, thus reducing operational costs and minimizing the environmental impact. 3) Reduced energy consumption: The use of enzymes and microorganisms can reduce the energy consumption of the process, making it more sustainable. 4) Enhanced enzymatic hydrolysis. 5) Reduced formation of inhibitory compounds. 6) Reduced equipment corrosion. 7) Increased sugar recovery.	1) High operating costs. 2) Limited feedstock flexibility. 3) Sensitivity to operating conditions. 4) Potential microbial contamination. 5) Complex process integration. 6) Lignin valorization.	Ibrahim et al. (2021); Muñoz et al. (2007); Monrroy et al. (2010); Itoh et al. (2003)
Biological + Dilute Acid (Bio-DA)	1) Reduced acid consumption. 2) Enhanced enzymatic hydrolysis. 3) Reduced formation of inhibitory compounds. 4) Low energy consumption. 5) Improved lignin quality.	1) Microbial contamination. 2) Sensitivity to operating conditions. 3) Limited feedstock flexibility. 4) Potential nutrient requirements. 5) Long process time.	Masami et al. (2008); Ma et al. (2010)
Biological + Steam Explosion (Bio-SExp.)	1) Reduced energy consumption. 2) Enhanced enzymatic hydrolysis. 3) Reduced formation of inhibitory compounds. 4) Improved lignin quality. 5) Low operating cost.	1) Microbial contamination. 2) Sensitivity to operating conditions. 3) Limited feedstock flexibility. 4) Potential nutrient requirements. 5) Long process time.	Meenakshisundaram et al. (2021); Li and Chen (2014); Taniguchi et al. (2010)

Microwave-Assisted Alkaline (MW-ALK)	1) High selectivity. 2) Faster process. 3) Reduced chemical consumption. 4) Improved enzymatic hydrolysis. 5) Enhanced energy efficiency.	1) Uneven heating. 2) Potential degradation of lignocellulosic materials. 3) Limited scalability. 4) Potential safety hazards. 5) Complex process.	Gazliya and Aparna (2021); Kamalini et al. (2018); Zhu et al. (2006)
Dilute Acid + Microwave (DA-MW)	1) High efficiency. 2) Low chemical consumption. 3) Shorter reaction time. 4) Improved yields.	1) High equipment and operating costs. 2) Energy-intensive. 3) Acid neutralization. 4) Scale-up challenges.	Qian et al. (2021); Mikulski and Kłosowski (2020)
Ionic Liquid + Ultrasonic (IL-UL)	1) High selectivity. 2) Low chemical consumption. 3) Shorter reaction time. 4) Improved yields.	1) High equipment and operating costs. 2) High energy consumption. 3) Limited scalability. 4) Environmental concerns.	Zhang et al. (2020); Yan et al. (2020)

1. Camelina straw treated by *T. versicolor* m4D

```
Parameters:
       Estimate Std. Error t value               Pr(>|t|)
mumax  1.004727  0.265426    3.79                0.00074 ***
Ks     0.681896  0.329231    2.07                0.04767 *
Xm     0.051894  0.000375  138.50 < 0.0000000000000002 ***
k1     0.007592  0.000120   63.07 < 0.0000000000000002 ***
k2    -0.042849  0.001100  -38.94 < 0.0000000000000002 ***
Y_xs   0.179877  0.002261   79.54 < 0.0000000000000002 ***
m_s    0.085736  0.022940    3.74                0.00085 ***
k_LD   0.528422  0.210965    2.50                0.01835 *
---
Signif. codes:  0 ‘***’ 0.001 ‘**’ 0.01 ‘*’ 0.05 ‘.’ 0.1 ‘ ’ 1

Residual standard error: 0.000896 on 28 degrees of freedom
Number of iterations to termination: 7
```

Fig. S1 Estimated parameters of growth kinetics.

```
Parameters:
     Estimate Std. Error t value              Pr(>|t|)
Y_xo   0.0798     0.0329    2.42                 0.022 *
m_o    9.5251     0.5133   18.56 <0.0000000000000002 ***
---
Signif. codes:  0 ‘***’ 0.001 ‘**’ 0.01 ‘*’ 0.05 ‘.’ 0.1 ‘ ’ 1

Residual standard error: 0.136 on 30 degrees of freedom
Number of iterations to termination: 11
```

Fig. S2 Estimated parameters of OUR.

```
> confint(fitval)
          2.5 %   97.5 %
mumax  0.48450  1.52495
Ks     0.03662  1.32718
Xm     0.05116  0.05263
k1     0.00736  0.00783
k2    -0.04501 -0.04069
Y_xs   0.17544  0.18431
m_s    0.04077  0.13070
k_LD   0.11494  0.94191
```

Fig. S3 Confidence interval (CI) of the estimated parameters of growth kinetics.

```
> confint(fitval_OUR)
      2.5 % 97.5 %
Y_xo 0.0153  0.144
m_o  8.5190 10.531
```

Fig. S4 Confidence interval (CI) of the estimated parameters of OUR.

```
Parameters:
     Estimate Std. Error t value            Pr(>|t|)
Y_cx   20.394      4.928    4.14             0.00026 ***
m_c     6.886      0.489   14.08 0.0000000000000093 ***
---
Signif. codes:  0 ‘***’ 0.001 ‘**’ 0.01 ‘*’ 0.05 ‘.’ 0.1 ‘ ’ 1

Residual standard error: 0.13 on 30 degrees of freedom
Number of iterations to termination: 2
```

Fig. S5 Estimated parameters of CPR.

```
Parameters:
      Estimate Std. Error t value Pr(>|t|)
alpha  0.08884    0.02899    3.06  0.00458 **
beta   0.01137    0.00288    3.95  0.00044 ***
---
Signif. codes:  0 ‘***’ 0.001 ‘**’ 0.01 ‘*’ 0.05 ‘.’ 0.1 ‘ ’ 1

Residual standard error: 0.000763 on 30 degrees of freedom
Number of iterations to termination: 2
```

Fig. S6 Estimated parameters of enzyme formation (P).

```
> confint(fitval_CPR)
     2.5 % 97.5 %
Y_cx 10.74  30.05
m_c   5.93   7.84
```

Fig. S7 Confidence interval (CI) of the estimated parameters of CPR.

```
> confint(fitval)
         2.5 %   97.5 %
alpha 0.032023 0.14567
beta  0.005729 0.01701
```

Fig. S8 Confidence interval (CI) of the estimated parameters of enzyme formation (P).

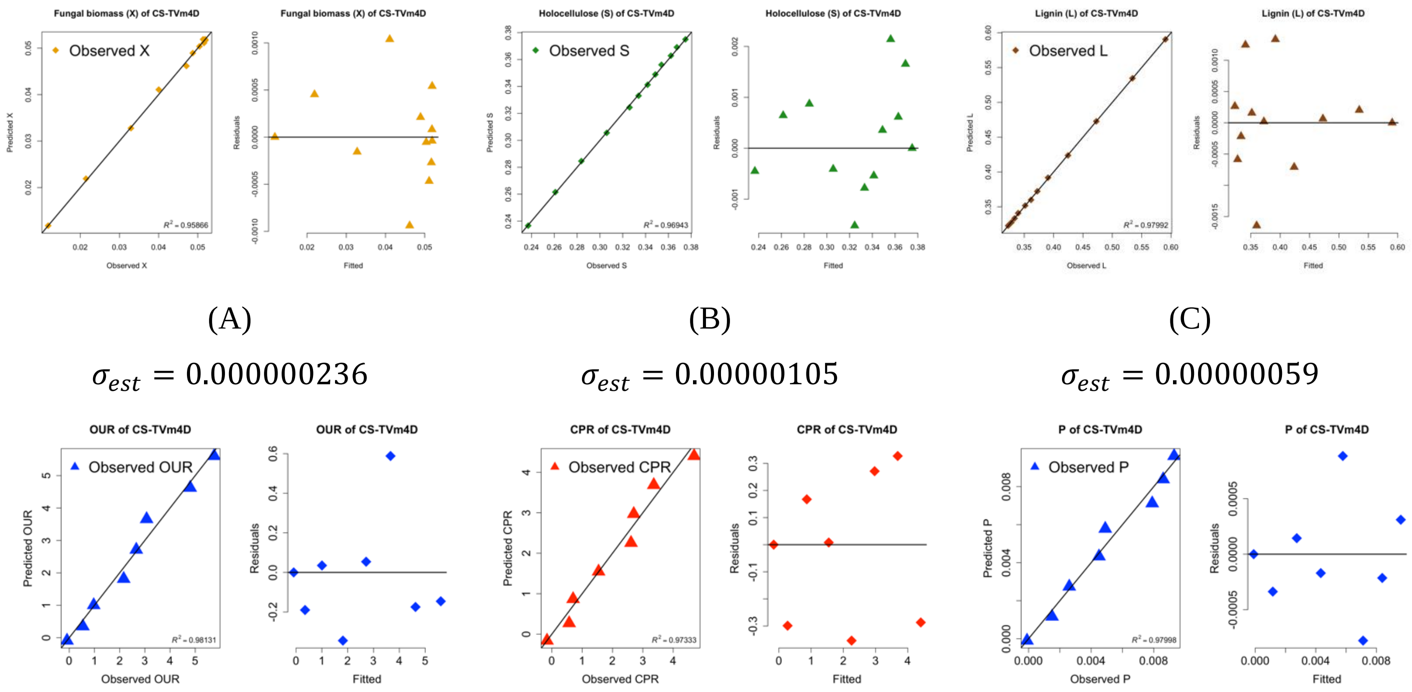

(A) $\sigma_{est} = 0.000000236$

(B) $\sigma_{est} = 0.00000105$

(C) $\sigma_{est} = 0.00000059$

(D) $\sigma_{est} = 0.0696$

(E) $\sigma_{est} = 0.0631$

(F) $\sigma_{est} = 0.0000002117$

Fig. S9 Model's normality check

2. Camelina straw treated by *T. versicolor* 52J

```
Parameters:
       Estimate Std. Error t value               Pr(>|t|)
mumax  1.210237   0.278331    4.35                0.00016 ***
Ks     0.663611   0.276646    2.40                0.02335 *
Xm     0.083876   0.000662  126.75 < 0.0000000000000002 ***
k1     0.004918   0.000178   27.64 < 0.0000000000000002 ***
k2    -0.052833   0.002352  -22.46 < 0.0000000000000002 ***
Y_xs   0.249325   0.003683   67.70 < 0.0000000000000002 ***
m_s    0.080692   0.013840    5.83              0.0000029 ***
k_LD   0.787283   0.237753    3.31                0.00256 **
---
Signif. codes:  0 ‘***’ 0.001 ‘**’ 0.01 ‘*’ 0.05 ‘.’ 0.1 ‘ ’ 1

Residual standard error: 0.00154 on 28 degrees of freedom
Number of iterations to termination: 6
```

Fig. S10 Estimated parameters of growth kinetics.

```
Parameters:
     Estimate Std. Error t value             Pr(>|t|)
Y_xo  0.01758    0.00196    8.98        0.00000000052 ***
m_o  20.56689    0.74081   27.76 < 0.0000000000000002 ***
---
Signif. codes:  0 ‘***’ 0.001 ‘**’ 0.01 ‘*’ 0.05 ‘.’ 0.1 ‘ ’ 1

Residual standard error: 0.317 on 30 degrees of freedom
Number of iterations to termination: 5
```

Fig. S11 Estimated parameters of OUR.

```
> confint(fitval)
          2.5 %    97.5 %
mumax  0.66472  1.755755
Ks     0.12139  1.205828
Xm     0.08258  0.085173
k1     0.00457  0.005267
k2    -0.05744 -0.048223
Y_xs   0.24211  0.256544
m_s    0.05357  0.107819
k_LD   0.32130  1.253270
```

Fig. S12 Confidence interval (CI) of the estimated parameters of growth kinetics.

```
> confint(fitval_OUR)
        2.5 %   97.5 %
Y_xo  0.01374  0.02141
m_o  19.11493 22.01885
```

Fig. S13 Confidence interval (CI) of the estimated parameters of OUR.

```
Parameters:
     Estimate Std. Error t value             Pr(>|t|)
Y_cx   26.714      2.083    12.8      0.0000000000001 ***
m_c    17.091      0.244    70.2 < 0.0000000000000002 ***
---
Signif. codes:  0 ‘***’ 0.001 ‘**’ 0.01 ‘*’ 0.05 ‘.’ 0.1 ‘ ’ 1

Residual standard error: 0.104 on 30 degrees of freedom
Number of iterations to termination: 3
```

Fig. S14 Estimated parameters of CPR.

```
Parameters:
      Estimate Std. Error t value    Pr(>|t|)
alpha  0.15392    0.01998     7.7 0.000000014 ***
beta   0.00537    0.00234     2.3       0.029 *
---
Signif. codes:  0 ‘***’ 0.001 ‘**’ 0.01 ‘*’ 0.05 ‘.’ 0.1 ‘ ’ 1

Residual standard error: 0.001 on 30 degrees of freedom
Number of iterations to termination: 2
```

Fig. S15 Estimated parameters of enzyme formation (P).

```
> confint(fitval_CPR)
     2.5 % 97.5 %
Y_cx 22.63  30.80
m_c  16.61  17.57
```

Fig. S16 Confidence interval (CI) of the estimated parameters of CPR.

```
> confint(fitval)
          2.5 %   97.5 %
alpha 0.1147487 0.193088
beta  0.0007867 0.009952
```

Fig. S17 Confidence interval (CI) of the estimated parameters of enzyme formation (P).

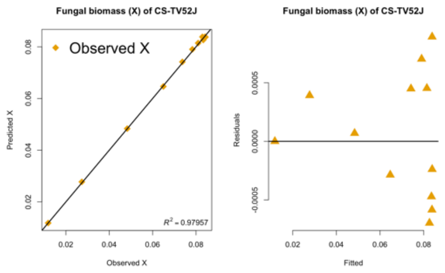

(A)

$\sigma_{est} = 0.0000002538$

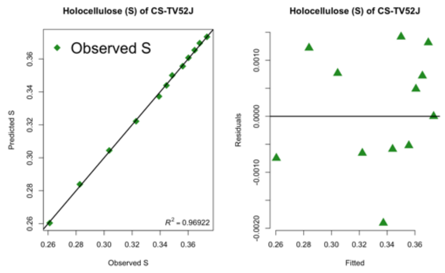

(B)

$\sigma_{est} = 0.0000009853$

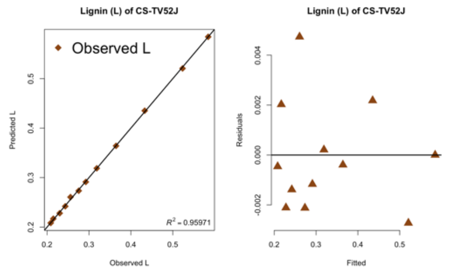

(C)

$\sigma_{est} = 0.000004278$

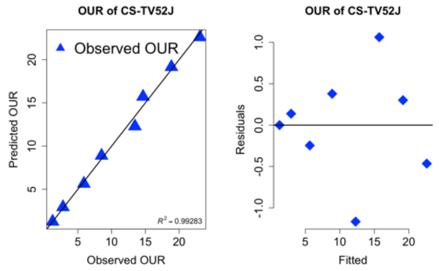

(D)

$\sigma_{est} = 0.3773$

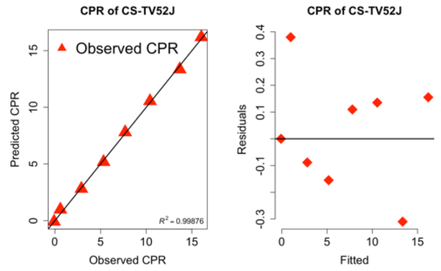

(E)

$\sigma_{est} = 0.04081$

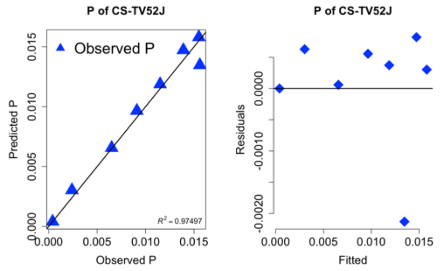

(F)

$\sigma_{est} = 0.0000007701$

Fig. S18 Model's normality check

3. Camelina treated by *P. chrysosporium*

```
Parameters:
       Estimate Std. Error t value              Pr(>|t|)    
mumax  1.005290   0.171238    5.87             0.0000026 ***
Ks     0.415143   0.162757    2.55                0.0165 *  
Xm     0.069888   0.000466  150.06 < 0.0000000000000002 ***
k1     0.002634   0.000126   20.91 < 0.0000000000000002 ***
k2    -0.057398   0.002986  -19.22 < 0.0000000000000002 ***
Y_xs   0.213295   0.002696   79.12 < 0.0000000000000002 ***
m_s    0.058504   0.010036    5.83             0.0000029 ***
k_LD   0.744440   0.225498    3.30                0.0026 ** 
---
Signif. codes:  0 ‘***’ 0.001 ‘**’ 0.01 ‘*’ 0.05 ‘.’ 0.1 ‘ ’ 1

Residual standard error: 0.00121 on 28 degrees of freedom
Number of iterations to termination: 7
```

Fig. S19 Estimated parameters of growth kinetics.

```
Parameters:
     Estimate Std. Error t value              Pr(>|t|)    
Y_xo  0.03652    0.00694    5.26              0.000011 ***
m_o  13.04558    0.57665   22.62 < 0.0000000000000002 ***
---
Signif. codes:  0 ‘***’ 0.001 ‘**’ 0.01 ‘*’ 0.05 ‘.’ 0.1 ‘ ’ 1

Residual standard error: 0.23 on 30 degrees of freedom
Number of iterations to termination: 6
```

Fig. S20 Estimated parameters of OUR.

```
> confint(fitval)
           2.5 %    97.5 %
mumax  0.669671  1.340910
Ks     0.096145  0.734142
Xm     0.068975  0.070801
k1     0.002387  0.002881
k2    -0.063250 -0.051546
Y_xs   0.208012  0.218579
m_s    0.038834  0.078174
k_LD   0.302473  1.186408
```

Fig. S21 Confidence interval (CI) of the estimated parameters of growth kinetics.

```
> confint(fitval_OUR)
        2.5 %   97.5 %
Y_xo  0.02293  0.05012
m_o  11.91536 14.17580
```

Fig. S22 Confidence interval (CI) of the estimated parameters of OUR.

```
Parameters:
     Estimate Std. Error t value              Pr(>|t|)    
Y_cx   16.055      2.982    5.38             0.0000079 ***
m_c     8.983      0.331   27.17 < 0.0000000000000002 ***
---
Signif. codes:  0 ‘***’ 0.001 ‘**’ 0.01 ‘*’ 0.05 ‘.’ 0.1 ‘ ’ 1

Residual standard error: 0.132 on 30 degrees of freedom
Number of iterations to termination: 2
```

Fig. S23 Estimated parameters of CPR.

```
Parameters:
      Estimate Std. Error t value Pr(>|t|)    
alpha  0.10533    0.02334    4.51 0.000092 ***
beta   0.00900    0.00259    3.48   0.0016 ** 
---
Signif. codes:  0 ‘***’ 0.001 ‘**’ 0.01 ‘*’ 0.05 ‘.’ 0.1 ‘ ’ 1

Residual standard error: 0.00103 on 30 degrees of freedom
Number of iterations to termination: 3
```

Fig. S24 Estimated parameters of enzyme formation (P).

```
> confint(fitval_CPR)
      2.5 % 97.5 %
Y_cx 10.211 21.899
m_c   8.335  9.631
```

Fig. S25 Confidence interval (CI) of the estimated parameters of CPR.

```
> confint(fitval)
        2.5 %  97.5 %
alpha 0.05958 0.15108
beta  0.00393 0.01407
```

Fig. S26 Confidence interval (CI) of the estimated parameters of enzyme formation (P).

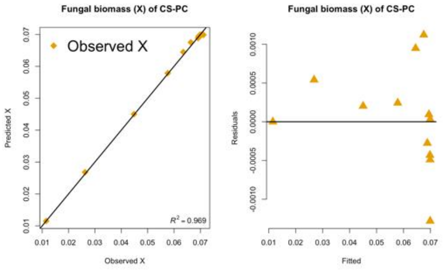

(A)

$\sigma_{est} = 0.0000003918$

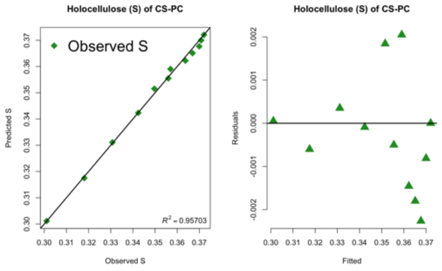

(B)

$\sigma_{est} = 0.000001623$

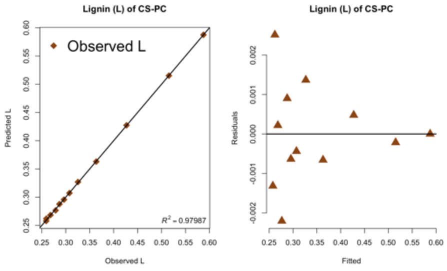

(C)

$\sigma_{est} = 0.000001411$

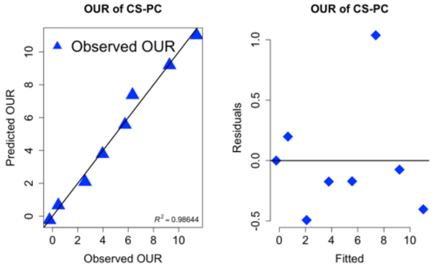

(D)

$\sigma_{est} = 0.1982$

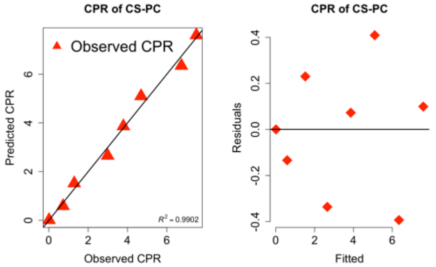

(E)

$\sigma_{est} = 0.06511$

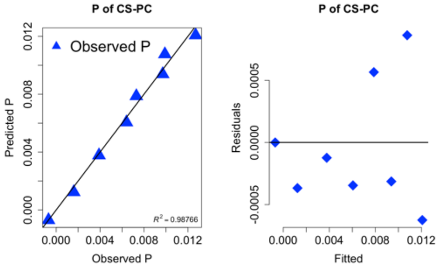

(F)

$\sigma_{est} = 0.000000228$

Fig. S27 Model's normality check

4. Switchgrass treated by *T. versicolor* m4D

```
Parameters:
       Estimate Std. Error t value              Pr(>|t|)
mumax  0.911432   0.216010    4.22               0.00023 ***
Ks     0.689650   0.315263    2.19               0.03721 *
Xm     0.047313   0.000318  148.96 < 0.0000000000000002 ***
k1     0.004629   0.000137   33.75 < 0.0000000000000002 ***
k2    -0.043330   0.002004  -21.62 < 0.0000000000000002 ***
Y_xs   0.152444   0.002016   75.62 < 0.0000000000000002 ***
m_s    0.100053   0.018497    5.41             0.0000091 ***
k_LD   1.037220   0.315740    3.29               0.00274 **
---
Signif. codes:  0 ‘***’ 0.001 ‘**’ 0.01 ‘*’ 0.05 ‘.’ 0.1 ‘ ’ 1

Residual standard error: 0.000776 on 28 degrees of freedom
Number of iterations to termination: 8
```

Fig. S28 Estimated parameters of growth kinetics.

```
Parameters:
     Estimate Std. Error t value              Pr(>|t|)
Y_xo   0.0780     0.0129    6.05            0.00000049 ***
m_o    9.7117     0.1391   69.81 < 0.0000000000000002 ***
---
Signif. codes:  0 ‘***’ 0.001 ‘**’ 0.01 ‘*’ 0.05 ‘.’ 0.1 ‘ ’ 1

Residual standard error: 0.0796 on 38 degrees of freedom
Number of iterations to termination: 4
```

Fig. S29 Estimated parameters of OUR.

```
> confint(fitval)
          2.5 %    97.5 %
mumax  0.488060  1.334804
Ks     0.071747  1.307554
Xm     0.046691  0.047936
k1     0.004361  0.004898
k2    -0.047257 -0.039402
Y_xs   0.148493  0.156395
m_s    0.063799  0.136307
k_LD   0.418381  1.656059
```

Fig. S30 Confidence interval (CI) of the estimated parameters of growth kinetics.

```
> confint(fitval_OUR)
        2.5 % 97.5 %
Y_xo 0.05269 0.1032
m_o  9.43903 9.9844
```

Fig. S31 Confidence interval (CI) of the estimated parameters of OUR.

```
Parameters:
     Estimate Std. Error t value              Pr(>|t|)
Y_cx   10.300      2.581    3.99               0.00029 ***
m_c     5.517      0.169   32.59 < 0.0000000000000002 ***
---
Signif. codes:  0 ‘***’ 0.001 ‘**’ 0.01 ‘*’ 0.05 ‘.’ 0.1 ‘ ’ 1

Residual standard error: 0.0969 on 38 degrees of freedom
Number of iterations to termination: 3
```

Fig. S32 Estimated parameters of CPR.

```
Parameters:
      Estimate Std. Error t value Pr(>|t|)
alpha  0.12378    0.03380    3.66  0.00076 ***
beta   0.00916    0.00222    4.13  0.00019 ***
---
Signif. codes:  0 ‘***’ 0.001 ‘**’ 0.01 ‘*’ 0.05 ‘.’ 0.1 ‘ ’ 1

Residual standard error: 0.00127 on 38 degrees of freedom
Number of iterations to termination: 2
```

Fig. S33 Estimated parameters of enzyme formation (P).

```
> confint(fitval_CPR)
     2.5 % 97.5 %
Y_cx 5.240 15.359
m_c  5.186  5.849
```

Fig. S34 Confidence interval (CI) of the estimated parameters of CPR.

```
> confint(fitval)
          2.5 %   97.5 %
alpha 0.057541 0.19003
beta  0.004817 0.01351
```

Fig. S35 Confidence interval (CI) of the estimated parameters of enzyme formation (P).

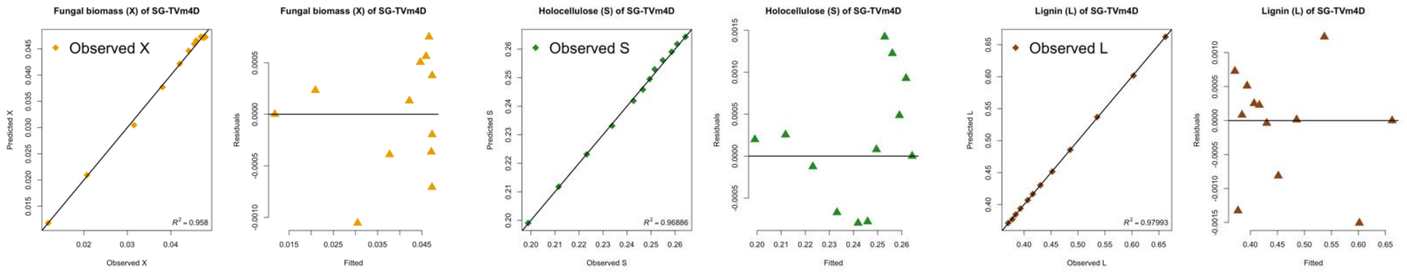

(A) $\sigma_{est} = 0.0000002739$

(B) $\sigma_{est} = 0.0000005365$

(C) $\sigma_{est} = 0.0000005944$

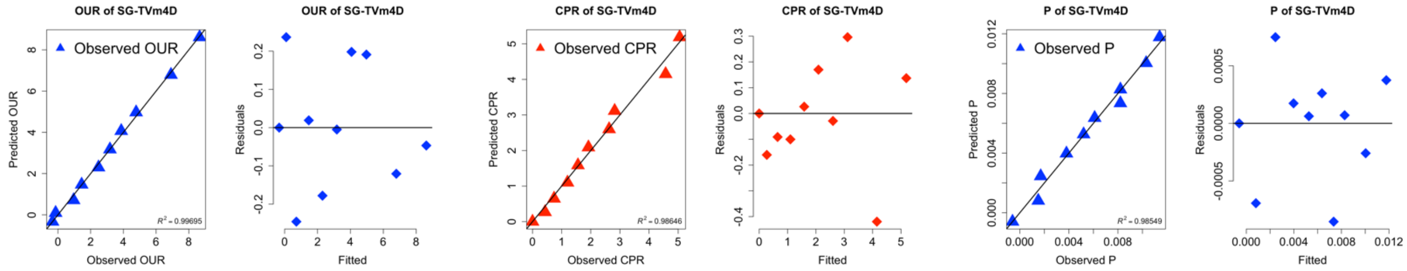

(D) $\sigma_{est} = 0.0241$

(E) $\sigma_{est} = 0.03569$

(F) $\sigma_{est} = 0.0000002085$

Fig. S36 Model's normality check

5. Switchgrass treated by *T. versicolor* 52J

```
Parameters:
       Estimate Std. Error t value             Pr(>|t|)
mumax  1.215575   0.162625    7.47       0.000000038490 ***
Ks     0.538461   0.152983    3.52               0.0015 **
Xm     0.093987   0.000335  280.61 < 0.0000000000000002 ***
k1     0.002872   0.000156   18.43 < 0.0000000000000002 ***
k2    -0.037405   0.003727  -10.04       0.000000000089 ***
Y_xs   0.295693   0.002258  130.97 < 0.0000000000000002 ***
m_s    0.084596   0.007979   10.60       0.000000000026 ***
k_LD   2.961811   0.546861    5.42       0.000008904434 ***
---
Signif. codes:  0 ‘***’ 0.001 ‘**’ 0.01 ‘*’ 0.05 ‘.’ 0.1 ‘ ’ 1

Residual standard error: 0.000881 on 28 degrees of freedom
Number of iterations to termination: 7
```

Fig. S37 Estimated parameters of growth kinetics.

```
Parameters:
     Estimate Std. Error t value            Pr(>|t|)
Y_xo  0.00921    0.00107    8.59 0.000000001401559 ***
m_o  19.43401    1.50222   12.94 0.000000000000083 ***
---
Signif. codes:  0 ‘***’ 0.001 ‘**’ 0.01 ‘*’ 0.05 ‘.’ 0.1 ‘ ’ 1

Residual standard error: 0.832 on 30 degrees of freedom
Number of iterations to termination: 4
```

Fig. S38 Estimated parameters of OUR.

```
> confint(fitval)
          2.5 %    97.5 %
mumax  0.896835  1.534315
Ks     0.238620  0.838303
Xm     0.093330  0.094643
k1     0.002567  0.003177
k2    -0.044709 -0.030101
Y_xs   0.291268  0.300118
m_s    0.068957  0.100235
k_LD   1.889983  4.033639
```

Fig. S39 Confidence interval (CI) of the estimated parameters of growth kinetics.

```
> confint(fitval_OUR)
         2.5 %   97.5 %
Y_xo  0.007111  0.01132
m_o  16.489722 22.37830
```

Fig. S40 Confidence interval (CI) of the estimated parameters of OUR.

```
Parameters:
     Estimate Std. Error t value             Pr(>|t|)
Y_cx   19.904      2.346    8.48         0.0000000018 ***
m_c    18.094      0.279   64.89 < 0.0000000000000002 ***
---
Signif. codes:  0 ‘***’ 0.001 ‘**’ 0.01 ‘*’ 0.05 ‘.’ 0.1 ‘ ’ 1

Residual standard error: 0.154 on 30 degrees of freedom
Number of iterations to termination: 3
```

Fig. S41 Estimated parameters of CPR.

```
Parameters:
      Estimate Std. Error t value  Pr(>|t|)
alpha  0.12637    0.02195    5.76 0.0000028 ***
beta   0.00781    0.00261    2.99    0.0055 **
---
Signif. codes:  0 ‘***’ 0.001 ‘**’ 0.01 ‘*’ 0.05 ‘.’ 0.1 ‘ ’ 1

Residual standard error: 0.00144 on 30 degrees of freedom
Number of iterations to termination: 2
```

Fig. S42 Estimated parameters of enzyme formation (P).

```
> confint(fitval_CPR)
     2.5 % 97.5 %
Y_cx 15.31  24.50
m_c  17.55  18.64
```

Fig. S43 Confidence interval (CI) of the estimated parameters of CPR.

```
> confint(fitval)
         2.5 %  97.5 %
alpha 0.083351 0.16939
beta  0.002698 0.01293
```

Fig. S44 Confidence interval (CI) of the estimated parameters of enzyme formation (P).

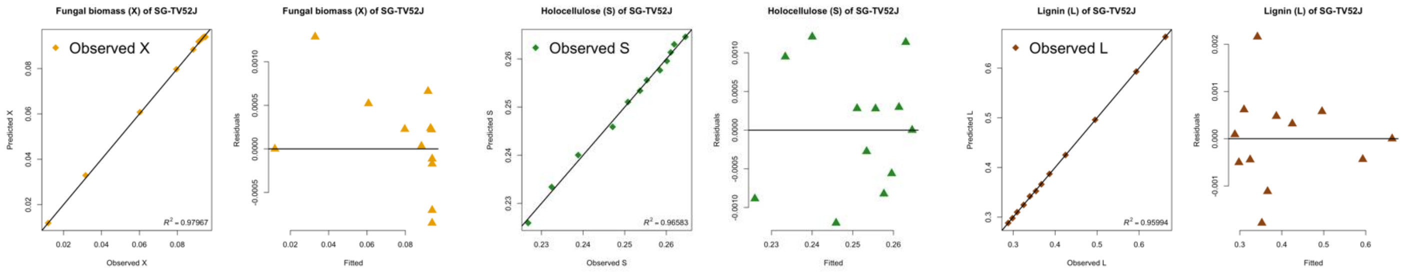

(A) $\sigma_{est} = 0.0000003149$

(B) $\sigma_{est} = 0.0000005986$

(C) $\sigma_{est} = 0.0000008979$

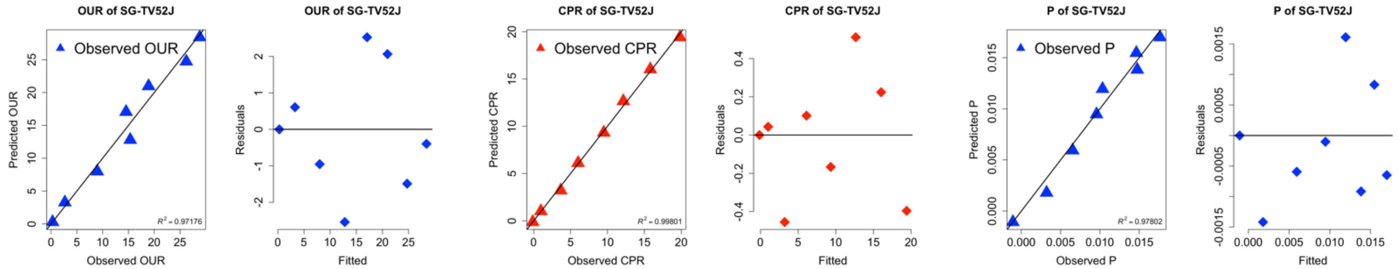

(D) $\sigma_{est} = 2.596$

(E) $\sigma_{est} = 0.08942$

(F) $\sigma_{est} = 0.0000008654$

Fig. S45 Model’s normality check

6. Switchgrass treated by *P. chrysosporium*

```
Parameters:
       Estimate Std. Error t value              Pr(>|t|)
mumax  0.787969  0.153964    5.12       0.000020093960 ***
Ks     0.417903  0.201279    2.08               0.0472 *
Xm     0.072919  0.000516  141.38 < 0.0000000000000002 ***
k1     0.002593  0.000245   10.58       0.000000000027 ***
k2    -0.025197  0.006914   -3.64               0.0011 **
Y_xs   0.246544  0.002393  103.03 < 0.0000000000000002 ***
m_s    0.045728  0.020449    2.24               0.0335 *
k_LD  -0.057509  1.343714   -0.04               0.9662
---
Signif. codes:  0 ‘***’ 0.001 ‘**’ 0.01 ‘*’ 0.05 ‘.’ 0.1 ‘ ’ 1

Residual standard error: 0.00123 on 28 degrees of freedom
Number of iterations to termination: 12
```

Fig. S46 Estimated parameters of growth kinetics.

```
Parameters:
     Estimate Std. Error t value             Pr(>|t|)
Y_xo  0.03113    0.00839    3.71              0.00074 ***
m_o  13.52574    0.81184   16.66 < 0.0000000000000002 ***
---
Signif. codes:  0 ‘***’ 0.001 ‘**’ 0.01 ‘*’ 0.05 ‘.’ 0.1 ‘ ’ 1

Residual standard error: 0.47 on 34 degrees of freedom
Number of iterations to termination: 6
```

Fig. S47 Estimated parameters of OUR.

```
> confint(fitval)
           2.5 %    97.5 %
mumax  0.486204  1.089734
Ks     0.023403  0.812404
Xm     0.071909  0.073930
k1     0.002113  0.003073
k2    -0.038747 -0.011646
Y_xs   0.241855  0.251234
m_s    0.005649  0.085807
k_LD  -2.691140  2.576122
```

Fig. S48 Confidence interval (CI) of the estimated parameters of growth kinetics.

```
> confint(fitval_OUR)
        2.5 %    97.5 %
Y_xo  0.01469  0.04758
m_o  11.93456 15.11692
```

Fig. S49 Confidence interval (CI) of the estimated parameters of OUR.

```
Parameters:
     Estimate Std. Error t value             Pr(>|t|)
Y_cx   22.109      2.516    8.79        0.00000000029 ***
m_c     8.998      0.236   38.14 < 0.0000000000000002 ***
---
Signif. codes:  0 ‘***’ 0.001 ‘**’ 0.01 ‘*’ 0.05 ‘.’ 0.1 ‘ ’ 1

Residual standard error: 0.137 on 34 degrees of freedom
Number of iterations to termination: 2
```

Fig. S50 Estimated parameters of CPR.

```
Parameters:
      Estimate Std. Error t value  Pr(>|t|)
alpha  0.13945    0.02442    5.71 0.0000021 ***
beta   0.00783    0.00229    3.42    0.0016 **
---
Signif. codes:  0 ‘***’ 0.001 ‘**’ 0.01 ‘*’ 0.05 ‘.’ 0.1 ‘ ’ 1

Residual standard error: 0.00133 on 34 degrees of freedom
Number of iterations to termination: 2
```

Fig. S51 Estimated parameters of enzyme formation (P).

```
> confint(fitval_CPR)
      2.5 % 97.5 %
Y_cx 17.177 27.040
m_c   8.536  9.461
```

Fig. S52 Confidence interval (CI) of the estimated parameters of CPR.

```
> confint(fitval)
          2.5 %   97.5 %
alpha 0.091585 0.18732
beta  0.003342 0.01232
```

Fig. S53 Confidence interval (CI) of the estimated parameters of enzyme formation (P).

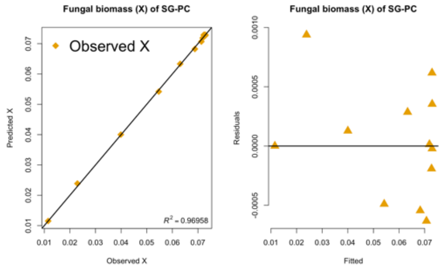

(A)

$\sigma_{est} = 0.0000002049$

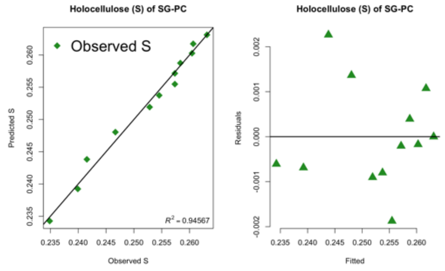

(B)

$\sigma_{est} = 0.000001183$

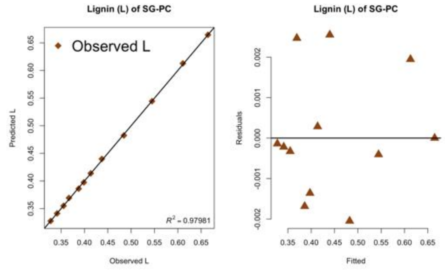

(C)

$\sigma_{est} = 0.000002143$

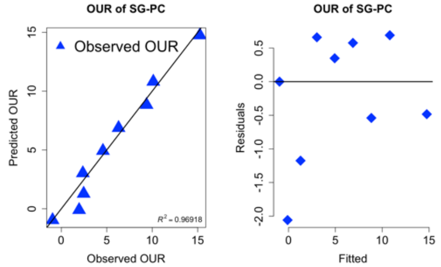

(D)

$\sigma_{est} = 0.8342$

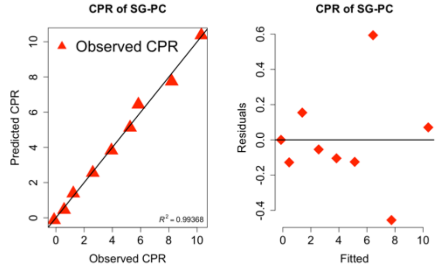

(E)

$\sigma_{est} = 0.07045$

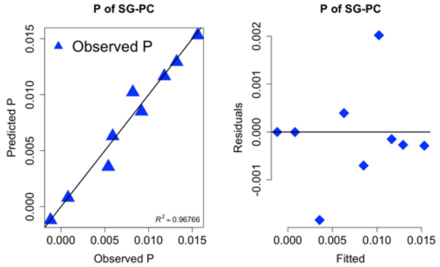

(F)

$\sigma_{est} = 0.0000009207$

Fig. S54 Model’s normality check

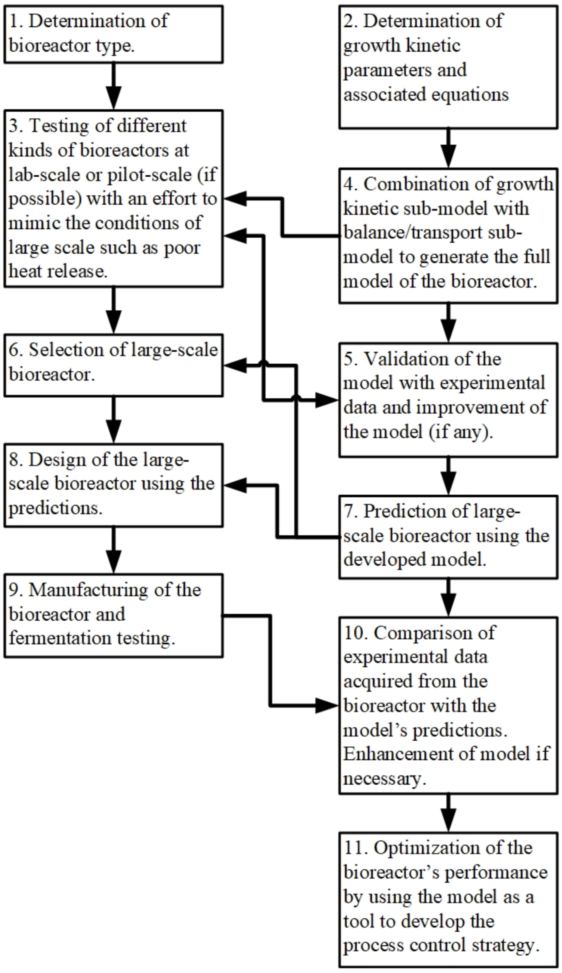

Figure S55: Details of the strategy for using models as tools in the design and optimization of the operation of SSF bioreactors.

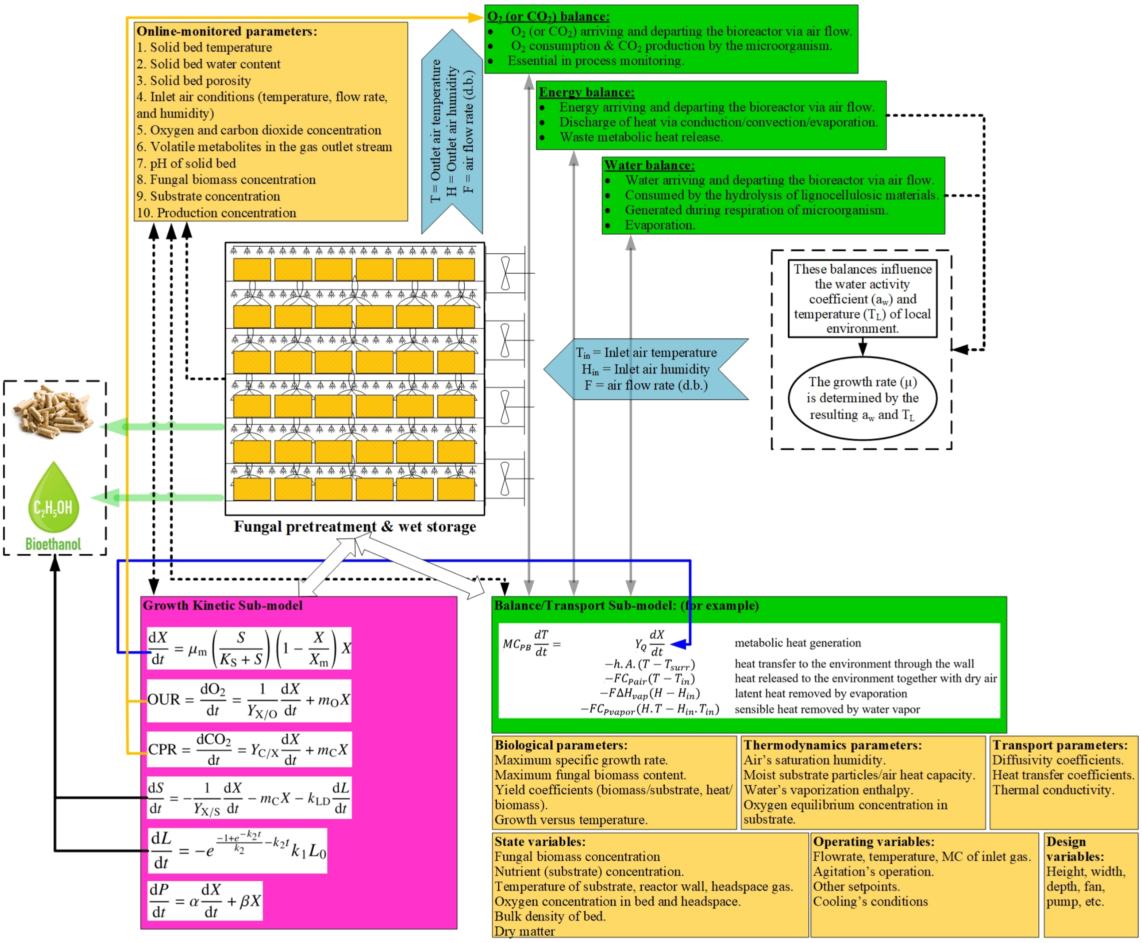

Figure S56: Roles of sub-models in development and optimization of SSF bioreactor.

REFERENCES

Ali, N., Hamouda, H. I., Su, H., Li, F.-L., and Lu, M. (2020). Combinations of alkaline hydrogen peroxide and lithium chloride/n, n-dimethylacetamide pretreatments of corn stalk for improved biomethanation. *Environmental Research* 186, 109563

Alinia, R., Zabihi, S., Esmaeilzadeh, F., and Kalajahi, J. F. (2010). Pretreatment of wheat straw by supercritical co2 and its enzymatic hydrolysis for sugar production. *Biosystems engineering* 107, 61–66

Auxenfans, T., Crônier, D., Chabbert, B., and Paës, G. (2017). Understanding the structural and chemical changes of plant biomass following steam explosion pretreatment. *Biotechnology for biofuels* 10, 1–16

Borand, M. N. and Karaosmanoğlu, F. (2018). Effects of organosolv pretreatment conditions for lignocellulosic biomass in biorefinery applications: a review. *Journal of renewable and sustainable energy* 10, 033104

Chen, W.-H., Nižetić, S., Sirohi, R., Huang, Z., Luque, R., Papadopoulos, A. M., et al. (2022). Liquid hot water as sustainable biomass pretreatment technique for bioenergy production: A review. *Bioresource technology* 344, 126207

Chen, W.-H., Pen, B.-L., Yu, C.-T., and Hwang, W.-S. (2011). Pretreatment efficiency and structural characterization of rice straw by an integrated process of dilute-acid and steam explosion for bioethanol production. *Bioresource technology* 102, 2916–2924

Chundawat, S. P., Pal, R. K., Zhao, C., Campbell, T., Teymouri, F., Videto, J., et al. (2020). Ammonia fiber expansion (afex) pretreatment of lignocellulosic biomass. *JoVE (Journal of Visualized Experiments)* , e57488

Duque, A., Manzanares, P., Ballesteros, I., and Ballesteros, M. (2016). Steam explosion as lignocellulosic biomass pretreatment. *Biomass fractionation technologies for a lignocellulosic feedstock based biorefinery* , 349–368

Emmel, A., Mathias, A. L., Wypych, F., and Ramos, L. P. (2003). Fractionation of eucalyptus grandis chips by dilute acid-catalysed steam explosion. *Bioresource technology* 86, 105–115

Gazliya, N. and Aparna, K. (2021). Microwave-assisted alkaline delignification of banana peduncle. *Journal of Natural Fibers* 18, 664–673

Huang, C., Li, R., Tang, W., Zheng, Y., and Meng, X. (2022). Improve enzymatic hydrolysis of lignocellulosic biomass by modifying lignin structure via sulfite pretreatment and using lignin blockers. *Fermentation* 8, 558

Ibrahim, Q., Lu-Chau, T. A., Eibes, G., and Kruse, A. (2021). Combination of fungal and organosolv pretreatment for the fractionation of beech wood into 2g sugars. *Available at SSRN 3965225*

Itoh, H., Wada, M., Honda, Y., Kuwahara, M., and Watanabe, T. (2003). Bioorganosolve pretreatments for simultaneous saccharification and fermentation of beech wood by ethanolysis and white rot fungi. *Journal of Biotechnology* 103, 273–280

Iyer, P. V., Wu, Z.-W., Kim, S. B., and Lee, Y. Y. (1996). Ammonia recycled percolation process for pretreatment of herbaceous biomass. *Applied biochemistry and biotechnology* 57, 121–132

Jing, X., Chai, X., Long, S., Liu, T., Si, M., Zheng, X., et al. (2022). Urea/sodium hydroxide pretreatments enhance decomposition of maize straw in soils and sorption of straw residues toward herbicides. *Journal of Hazardous Materials* 431, 128467

Kamalini, A., Muthusamy, S., Ramapriya, R., Muthusamy, B., and Pugazhendhi, A. (2018). Optimization of sugar recovery efficiency using microwave assisted alkaline pretreatment of cassava stem using response surface methodology and its structural characterization. *Journal of Molecular Liquids* 254, 55–63

Kan, T., Strezov, V., and Evans, T. J. (2016). Lignocellulosic biomass pyrolysis: A review of product properties and effects of pyrolysis parameters. *Renewable and sustainable energy reviews* 57, 1126–1140

Kang, K. E., Jeong, G.-T., Sunwoo, C., and Park, D.-H. (2012). Pretreatment of rapeseed straw by soaking in aqueous ammonia. *Bioprocess and biosystems engineering* 35, 77–84

Karthika, K., Arun, A., and Rekha, P. (2012). Enzymatic hydrolysis and characterization of lignocellulosic biomass exposed to electron beam irradiation. *Carbohydrate polymers* 90, 1038–1045

Kim, J. S., Lee, Y., and Kim, T. H. (2016). A review on alkaline pretreatment technology for bioconversion of lignocellulosic biomass. *Bioresource technology* 199, 42–48

Kim, S. B. and Lee, Y. (2002). Diffusion of sulfuric acid within lignocellulosic biomass particles and its impact on dilute-acid pretreatment. *Bioresource technology* 83, 165–171

Kim, T. H., Taylor, F., and Hicks, K. B. (2008). Bioethanol production from barley hull using saa (soaking in aqueous ammonia) pretreatment. *Bioresource technology* 99, 5694–5702

Kostas, E. T., Beneroso, D., and Robinson, J. P. (2017). The application of microwave heating in bioenergy: A review on the microwave pre-treatment and upgrading technologies for biomass. *Renewable and Sustainable Energy Reviews* 77, 12–27

Lau, M. W., Gunawan, C., and Dale, B. E. (2009). The impacts of pretreatment on the fermentability of pretreated lignocellulosic biomass: a comparative evaluation between ammonia fiber expansion and dilute acid pretreatment. *Biotechnology for biofuels* 2, 1–11

Lethesh, K. C., Evjen, S., Venkatraman, V., Shah, S. N., and Fiksdahl, A. (2020). Highly efficient cellulose dissolution by alkaline ionic liquids. *Carbohydrate polymers* 229, 115594

Li, G. and Chen, H. (2014). Synergistic mechanism of steam explosion combined with fungal treatment by phellinus baumii for the pretreatment of corn stalk. *Biomass and Bioenergy* 67, 1–7

Li, H., Qu, Y., Yang, Y., Chang, S., and Xu, J. (2016). Microwave irradiation–a green and efficient way to pretreat biomass. *Bioresource technology* 199, 34–41

Li, Q., Ji, G.-S., Tang, Y.-B., Gu, X.-D., Fei, J.-J., and Jiang, H.-Q. (2012). Ultrasound-assisted compatible in situ hydrolysis of sugarcane bagasse in cellulase-aqueous–n-methylmorpholine-n-oxide system for improved saccharification. *Bioresource Technology* 107, 251–257

Ma, F., Yang, N., Xu, C., Yu, H., Wu, J., and Zhang, X. (2010). Combination of biological pretreatment with mild acid pretreatment for enzymatic hydrolysis and ethanol production from water hyacinth. *Bioresource technology* 101, 9600–9604

Mafe, O. A., Davies, S. M., Hancock, J., and Du, C. (2015). Development of an estimation model for the evaluation of the energy requirement of dilute acid pretreatments of biomass. *biomass and bioenergy* 72, 28–38

Masami, G. O., Usui, I., and Urano, N. (2008). Ethanol production from the water hyacinth eichhornia crassipes by yeast isolated from various hydrospheres. *African journal of microbiology research* 2, 110–113

McIntosh, S., Zhang, Z., Palmer, J., Wong, H.-H., Doherty, W. O., and Vancov, T. (2016). Pilot-scale cellulosic ethanol production using eucalyptus biomass pre-treated by dilute acid and steam explosion. *Biofuels, Bioproducts and Biorefining* 10, 346–358

Meenakshisundaram, S., Fayeulle, A., Leonard, E., Ceballos, C., and Pauss, A. (2021). Fiber degradation and carbohydrate production by combined biological and chemical/physicochemical pretreatment methods of lignocellulosic biomass–a review. *Bioresource technology* 331, 125053

Mikulski, D. and Kłosowski, G. (2020). Microwave-assisted dilute acid pretreatment in bioethanol production from wheat and rye stillages. *Biomass and bioenergy* 136, 105528

Monrroy, M., Ibanez, J., Melin, V., Baeza, J., Mendonça, R. T., Contreras, D., et al. (2010). Bioorganosolv pretreatments of p. radiata by a brown rot fungus (gloephyllum trabeum) and ethanolysis. *Enzyme and Microbial Technology* 47, 11–16

Montero, I., Miranda, T., Sepúlveda, F. J., Arranz, J. I., and Nogales, S. (2014). Analysis of pelletizing of granulometric separation powder from cork industries. *Materials* 7, 6686–6700

Mood, S. H., Golfeshan, A. H., Tabatabaei, M., Jouzani, G. S., Najafi, G. H., Gholami, M., et al. (2013). Lignocellulosic biomass to bioethanol, a comprehensive review with a focus on pretreatment. *Renewable and Sustainable Energy Reviews* 27, 77–93

Muñoz, C., Mendonça, R., Baeza, J., Berlin, A., Saddler, J., and Freer, J. (2007). Bioethanol production from bio-organosolv pulps of pinus radiata and acacia dealbata. *Journal of Chemical Technology & Biotechnology: International Research in Process, Environmental & Clean Technology* 82, 767–774

Nguyen, T.-A. D., Kim, K.-R., Han, S. J., Cho, H. Y., Kim, J. W., Park, S. M., et al. (2010). Pretreatment of rice straw with ammonia and ionic liquid for lignocellulose conversion to fermentable sugars. *Bioresource Technology* 101, 7432–7438

Pandey, R., Nahar, N., Tumuluru, J. S., and Pryor, S. W. (2019). Quantifying reductions in soaking in aqueous ammonia pretreatment severity and enzymatic hydrolysis conditions for corn stover pellets. *Bioresource Technology Reports* 7, 100187

Qian, M., Lei, H., Villota, E., Zhao, Y., Wang, C., Huo, E., et al. (2021). High yield production of nanocrystalline cellulose by microwave-assisted dilute-acid pretreatment combined with enzymatic hydrolysis. *Chemical Engineering and Processing-Process Intensification* 160, 108292

Rosenau, T. and French, A. D. (2021). N-methylmorpholine-n-oxide (nmmo): hazards in practice and pitfalls in theory. *Cellulose* 28, 5985–5990

Sathitsuksanoh, N., Xu, B., Zhao, B., and Zhang, Y.-H. P. (2013). Overcoming biomass recalcitrance by combining genetically modified switchgrass and cellulose solvent-based lignocellulose pretreatment. *PLoS One* 8, e73523

Sathitsuksanoh, N., Zhu, Z., and Zhang, Y.-H. P. (2012). Cellulose solvent-and organic solvent-based lignocellulose fractionation enabled efficient sugar release from a variety of lignocellulosic feedstocks. *Bioresource technology* 117, 228–233

Shao, L., Chen, H., Li, Y., Li, J., Chen, G., and Wang, G. (2020). Pretreatment of corn stover via sodium hydroxide–urea solutions to improve the glucose yield. *Bioresource technology* 307, 123191

Sharma, H. K., Xu, C., and Qin, W. (2019). Biological pretreatment of lignocellulosic biomass for biofuels and bioproducts: an overview. *Waste and Biomass Valorization* 10, 235–251

Show, K.-Y., Yan, Y.-G., and Lee, D.-J. (2019). Algal biomass harvesting and drying. In *Biofuels from Algae* (Elsevier). 135–166

Su, Y., Yu, X., Sun, Y., Wang, G., Chen, H., and Chen, G. (2018). Evaluation of screened lignin-degrading fungi for the biological pretreatment of corn stover. *Scientific reports* 8, 1–11

Sung, Y. J. and Shin, S.-J. (2011). Compositional changes in industrial hemp biomass (cannabis sativa l.) induced by electron beam irradiation pretreatment. *biomass and bioenergy* 35, 3267–3270

Taniguchi, M., Takahashi, D., Watanabe, D., Sakai, K., Hoshino, K., Kouya, T., et al. (2010). Effect of steam explosion pretreatment on treatment with pleurotus ostreatus for the enzymatic hydrolysis of rice straw. *Journal of bioscience and bioengineering* 110, 449–452

Travaini, R., Martín-Juárez, J., Lorenzo-Hernando, A., and Bolado-Rodríguez, S. (2016). Ozonolysis: An advantageous pretreatment for lignocellulosic biomass revisited. *Bioresource Technology* 199, 2–12

Verma, M., Loha, C., Sinha, A. N., and Chatterjee, P. K. (2017). Drying of biomass for utilising in co-firing with coal and its impact on environment–a review. *Renewable and sustainable energy reviews* 71, 732–741

Wan, C. and Li, Y. (2012). Fungal pretreatment of lignocellulosic biomass. *Biotechnology advances* 30, 1447–1457

Wang, G., Pan, X., Zhu, J., Gleisner, R., and Rockwood, D. (2009). Sulfite pretreatment to overcome recalcitrance of lignocellulose (sporl) for robust enzymatic saccharification of hardwoods. *Biotechnology Progress* 25, 1086–1093

Xiao, R., Chen, X., Wang, F., and Yu, G. (2010). Pyrolysis pretreatment of biomass for entrained-flow gasification. *Applied Energy* 87, 149–155

Yan, J., Zhao, Y., Li, K., Zhang, H., Fan, L., and Lu, Z. (2020). Efficient production of biodiesel from ionic liquid catalyzed esterification using ultrasonic-microwave combined intensification. *Chemical Engineering and Processing-Process Intensification* 149, 107870

Yoon, H., Wu, Z., and Lee, Y. (1995). Ammonia-recycled percolation process for pretreatment of biomass feedstock. *Applied Biochemistry and Biotechnology* 51, 5–19

Zhang, C., Liu, R., Xiang, J., Kang, H., Liu, Z., and Huang, Y. (2014). Dissolution mechanism of cellulose in n, n-dimethylacetamide/lithium chloride: revisiting through molecular interactions. *The Journal of Physical Chemistry B* 118, 9507–9514

Zhang, M., Song, X., Pei, Z., and Wang, D. (2010). Effects of mechanical comminution on enzymatic conversion of cellulosic biomass in biofuel manufacturing: a review. In *International Manufacturing Science and Engineering Conference*. vol. 49460, 497–504

Zhang, Q., Chen, Q.-H., and He, G.-Q. (2020). Effect of ultrasonic-ionic liquid pretreatment on the hydrolysis degree and antigenicity of enzymatic hydrolysates from whey protein. *Ultrasonics Sonochemistry* 63, 104926

Zhang, Q., Hu, J., and Lee, D.-J. (2017). Pretreatment of biomass using ionic liquids: research updates. *Renewable Energy* 111, 77–84

Zhao, C., Shao, Q., and Chundawat, S. P. (2020). Recent advances on ammonia-based pretreatments of lignocellulosic biomass. *Bioresource Technology* 298, 122446

Zhou, Z., Lei, F., Li, P., and Jiang, J. (2018). Lignocellulosic biomass to biofuels and biochemicals: A comprehensive review with a focus on ethanol organosolv pretreatment technology. *Biotechnology and Bioengineering* 115, 2683–2702

Zhou, Z., Ouyang, D., Liu, D., and Zhao, X. (2022). Oxidative pretreatment of lignocellulosic biomass for enzymatic hydrolysis: progress and challenges. *Bioresource Technology* , 128208

Zhu, S., Wu, Y., Yu, Z., Chen, Q., Wu, G., Yu, F., et al. (2006). Microwave-assisted alkali pre-treatment of wheat straw and its enzymatic hydrolysis. *Biosystems Engineering* 94, 437–442

Zhu, Z., Sathitsuksanoh, N., Vinzant, T., Schell, D. J., McMillan, J. D., and Zhang, Y.-H. P. (2009). Comparative study of corn stover pretreated by dilute acid and cellulose solvent-based lignocellulose fractionation: Enzymatic hydrolysis, supramolecular structure, and substrate accessibility. *Biotechnology and bioengineering* 103, 715–724
